# Joint Tracking and Classification of Multiple Targets with Scattering Center Model and CBMeMBer Filter †

**DOI:** 10.3390/s20061679

**Published:** 2020-03-17

**Authors:** Ronghui Zhan, Liping Wang, Jun Zhang

**Affiliations:** Science and Technology on Automatic Target Recognition Laboratory, National University of Defense Technology, Changsha 410073, Hunan, China; wangliping17@nudt.edu.cn (L.W.); zhangjun@nudt.edu.cn (J.Z.)

**Keywords:** joint tracking and classification, scattering center model, high range resolution profile, CBMeMBer filter, sequential Monte Carlo

## Abstract

This paper deals with joint tracking and classification (JTC) of multiple targets based on scattering center model (SCM) and wideband radar observations. We first introduce an SCM-based JTC method, where the SCM is used to generate the predicted high range resolution profile (HRRP) with the information of the target aspect angle, and target classification is implemented through the data correlation of observed HRRP with predicted HRRPs. To solve the problem of multi-target JTC in the presence of clutter and detection uncertainty, we then integrate the SCM-based JTC method into the CBMeMBer filter framework, and derive a novel SCM-JTC-CBMeMBer filter with Bayesian theory. To further tackle the complex integrals’ calculation involved in targets state and class estimation, we finally provide the sequential Monte Carlo (SMC) implementation of the proposed SCM-JTC-CBMeMBer filter. The effectiveness of the presented multi-target JTC method is validated by simulation results under the application scenario of maritime ship surveillance.

## 1. Introduction

Traditionally, target tracking and target classification are treated as two independent problems, and they are usually solved separately. However, these two problems are closely related. For example, tracking affects classification by providing flight envelope information for different air target classes, while classification affects tracking via selecting appropriate class-dependent kinematic models. Therefore, a good classification may benefit tracking and vice versa. For this reason, the joint tracking and classification (JTC) method is receiving more and more attention.

By now, many JTC methods have been proposed [1,2,3,4,5,6,7,8,9,10], and these methods can roughly be divided into three categories. The first category is the most popular one and is dedicated to point targets. In this case, the resolution of the tracking sensor is very limited, and realization of target classification has to exploit attribute/identity sensor (such as electronic support measure) information or target dynamics (such as class-dependent maneuverability) [1,2,3,4,5].

The second category treats the target as an extended target, and the measurement of the target is modeled as the extent in down-range direction (length of the target) or both in down-range and lateral-range directions (i.e., size of the observed target contour), based on the assumption that the target has an ellipsoidal shape [6,7,8]. Targets are classified with the feature information of different length or size.

The third category treats the target as a rigid body, and the measurements are the target geometric shapes, which correspond to the projection of the target computer-aided design (CAD) models on the charge-coupled device (CCD) sensor [9,10]. Target classification is realized by image features.

For the wideband radar, the high range resolution profile (HRRP) serves as an important signature (feature) for target classification. However, as is known to all, the HRRP is very sensitive to target pose and the length (down-range extent) is not a stable feature in the dynamic environment, especially when the relative state between target and sensor changes rapidly. Moreover, the constraint of ideal ellipsoidal shape assumption imposed on the target limits the real application of the extent-based JTC methods. In view of the fact that the 3D scattering center model (3D-SCM) [11,12,13] is very convenient to create a classification feature according to the pose and sensor parameters, we proposed a novel SCM-based single-target JTC method in the conference paper [14]. The presented method exploited the 3D-SCM to predict the pose-dependent HRRP classification feature, together with the observation data of target’s bearing, range and HRRP, to jointly infer the target state and class.

This paper is an extension of the SCM-based JTC method in [14] to the multi-target scenario, where targets with different classes may appear or disappear in the surveillance area, and false alarm/missed detection may exist. For the treatment of multi-target tracking, there are generally two main methods, i.e., the data-association-based method [15,16,17] and the random finite set (RFS)-based method [18,19]. The data-association-based method involves explicit associations. For example, the joint probabilistic data association (JPDA) algorithm [15] weights all the observations by association probabilities, and the multiple hypotheses tracking (MHT) algorithm [17] propagates association hypothesis. However, with the increase of the considered target number, the data-association-based method will suffer from a large computational burden. The RFS-based method models the multi-target state and the observations as RFSs and can avoid the explicit data association. Compared with the data-association-based method, the RFS-based method propagates the posterior density of the multi-target state recursively by means of the multi-target Bayes filter, and can be implemented through approximation approaches with a lower computational load. Therefore, the RFS-based method can serve as a good alternative to implement multi-target JTC.

Due to the intractability of Bayesian multi-target filter, two main approximation approaches, i.e., moment approximation (such as the probability hypothesis density (PHD) filter [20] and the cardinalized PHD (CPHD) filter [21]) and posterior density approximation (such as the multi-target multi-Bernoulli (MeMBer) filter, the cardinality balanced CBMeMBer filter [22] and the labeled multi-Bernoulli filter [23], etc.) are proposed, and have been widely used to various fields such as image processing [24,25] and multi-sensor fusion [26,27,28]. These approximation filters involve multiple integrals, and the implementation of the filters mainly depends on the characteristic of the system model. Generally, the Gaussian mixture (GM) implementation is suitable for the Gaussian linear system. However, for the non-Gaussian nonlinear system, sequential Monte Carlo (SMC) implementation has to be considered [29,30,31,32].

To deal with the problem of multi-target JTC, the SCM-based JTC method is integrated into the CBMeMBer filter framework in this paper, and the resulting filter is called SCM-JTC-CBMeMBer. Additionally, consider the high nonlinearity of the kinematic (range and bearing) and feature (HRRP) observation models, the proposed filter is implemented via the SMC technique.

The rest of this paper is organized as follows. The system model and a brief review of the CBMeMBer filter are provided in Section 2. The SCM-based JTC method and the proposed SCM-JTC-CBMeMBer filter are described in Section 3. The details on SMC implementation of the SCM-JTC-CBMeMBer filter are given in Section 4, followed by the simulation results in Section 5. A conclusion closes the paper.

## 2. Background

In this section, the system model (including state model and sensor observation model) will first be introduced. Then, a brief review of the CBMeMBer filter is given.

### 2.1. System Model

#### 2.1.1. Target Motion Model

Without loss of generality, the considered target moves on the 2D plane with a nearly constant velocity, and the evolvement process of the target state is given as

(1) $$x_{k}=Fx_{k-1}+w_{k}$$

where $x_{k}={\left[x_{k}\;y_{k}\;{\dot{x}}_{k}\;{\dot{y}}_{k}\right]}^{T}$ represents target state including position component $pos_{k}={\left[x_{k}\;y_{k}\right]}^{T}$ and velocity component $vel_{k}={\left[{\dot{x}}_{k}\;{\dot{y}}_{k}\right]}^{T}$. The subscript k denotes sampling time and T is the sign for vector transpose. F is the state transition matrix. $w_{k}\sim N(w;0,Q)$ represents the multi-dimensional Gaussian process noise vector, where N(ζ;μ,Σ) denotes Gaussian function with variable ζ, mean μ and covariance matrix Σ. F and Q can be further written as

(2) $$F=\left[\begin{array}{cccc} 1 & 0 & t & 0\\ 0 & 1 & 0 & t\\ 0 & 0 & 1 & 0\\ 0 & 0 & 0 & 1 \end{array}\right],\;Q=q^{2}\left[\begin{array}{cccc} t^{4}/4 & 0 & t^{3}/2 & 0\\ 0 & t^{4}/4 & 0 & t^{3}/2\\ t^{3}/2 & 0 & t^{2} & 0\\ 0 & t^{3}/2 & 0 & t^{2} \end{array}\right]$$

where t is the sampling interval and q is the acceleration variance.

#### 2.1.2. Sensor Observation Model

The radar can provide range and bearing measurements of the target’s centroid, and the observation model of kinematic (position) measurement can be described by

(3) $$z_{k}=\left[\begin{array}{c} r_{k}\\ \beta_{k} \end{array}\right]=\left[\begin{array}{c} \sqrt{x_{k}^{2}+y_{k}^{2}}+v_{1,k}\\ \tan^{-1}(y_{k}/x_{k})+v_{2,k} \end{array}\right]=h(x_{k})+v_{k}$$

where h(⋅) is the kinematic observation function and $r_{k}$ and $\beta_{k}$ represent noisy measurements of target range and bearing at time k, respectively. $v_{k}={\left[v_{1,k}\;v_{2,k}\right]}^{T}$ denotes the corresponding zero-mean observation noise with covariance matrix $R=E[v_{k}v_{k}^{T}]=diag[\sigma_{r}^{2},\sigma_{\beta }^{2}]$.

**Figure 1:**
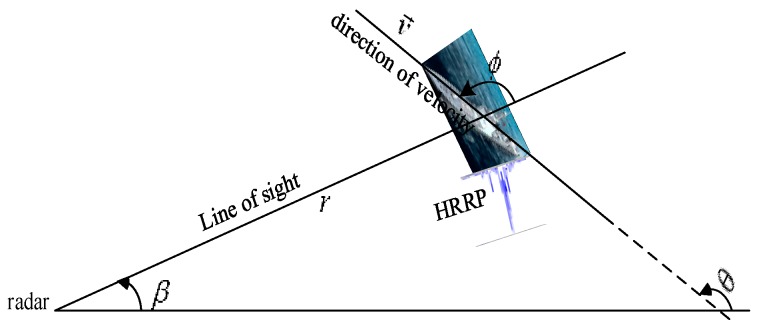
Illustration of target aspect angle.

As shown in Figure 1, for the low-speed maritime target, the heading is almost aligned with the axial direction of the target body because of its limited maneuverability. Under this condition, the aspect angle $\phi_{k}$ of the target can be obtained as

(4) $$\phi_{k}=\theta_{k}-\beta_{k}$$

where $\theta_{k}=\tan^{-1}({\dot{y}}_{k}/{\dot{x}}_{k})$ is the heading angle.

An equivalent expression of aspect angle is in the form of

(5) $$\phi_{k}=\cos^{-1}\left(\frac{\langle pos_{k},vel_{k}\rangle }{\left\|pos_{k}\right\|\cdot \left\|vel_{k}\right\|}\right)=\cos^{-1}\left(\frac{x_{k}{\dot{x}}_{k}+y_{k}{\dot{y}}_{k}}{\sqrt{x_{k}^{2}+y_{k}^{2}}\sqrt{{\dot{x}}_{k}^{2}+{\dot{y}}_{k}^{2}}}\right)$$

The 3D-SCM is an equivalent of the target in geometry space to the radar response in the electromagnetic field. It provides a concise and physically relevant description of the target’s scattering through a set of representative scattering parts, and thus a more effective way to characterize the target’s electromagnetic scattering behavior. The 3D-SCM consists of a set of scattering center with a specific position, amplitude and type parameters, and it can be represented by

(6) $$S={\left\{a_{n},\alpha_{n},x_{n},y_{n},z_{n}\right\}}_{n=1}^{N}$$

where $a_{n}$ is the amplitude of *n*th scattering center, $\alpha_{n}$ is a frequency-dependent factor, $\left(x_{n},y_{n},z_{n}\right)$ is the corresponding 3D spatial position in the target body coordinates and N is the number of scatters involved in the model. For a specific target class c, the associated 3D-SCM can be denoted as $S_{c}$.

The whole target’s backscattering with respect to radar instantaneous frequency f, viewing angles (i.e., azimuth angle ϕ and elevation angle γ) can be expressed as [13]

(7) $$E(f,\phi ,\gamma ,S)=\sum_{n=1}^{N}{\left(jf/f_{c}\right)}^{\alpha_{n}}a_{n}(\phi ,\gamma )\cdot \exp(-j4\pi (x_{n}\cos\gamma \cos\phi +y_{n}\cos\gamma \sin\phi +z_{n}\sin\gamma )/\lambda )$$

where λ is the wavelength, $f_{c}$ is the central frequency of signal, $j=\sqrt{-1}$ is the imaginary unit and $a_{n}(\phi ,\gamma )$ represents the amplitude of *n*th scattering center which may change with ϕ and γ.

At a specific viewing angle of (ϕ,γ), the corresponding projection position of the *n*th scattering center at the down-range direction is

(8) $$r_{n}(\phi ,\gamma )=x_{n}\cos\gamma \cos\phi +y_{n}\cos\gamma \sin\phi +z_{n}\sin\gamma$$

Assuming that the bandwidth of the radar signal is B and the *i*th discrete frequency point is

(9) $$f_{i}=f_{c}-B/2+i\cdot \Delta F,\;i=0,1,\cdots ,I$$

where ΔF is the frequency interval and I+1 is the total number of frequency points.

Then, the frequency response of the *i*th frequency point can be written as $E_{i}=E(f_{i},\phi ,\gamma ,S)$. After a direct operation of inverse discrete Fourier transform (IDFT) on frequency response sequence $E=[E_{0},E_{1},\cdots ,E_{I}]$, the desired HRRP can be immediately obtained.

When the target’s motion is restricted on the 2D plane, the observation model of HRRP is represented as

(10) $$\begin{array}{ll} d & =g(\phi ,S)+n\\ & =IDFT[E_{i}=E(f_{i},\phi ,\gamma ,S),i=0,1,\cdots ,I]+n\\ & =IDFT(E)+n \end{array}$$

where d denotes the (*I*+1)-dimensional measurement of the HRRP and each component of d corresponds to one range resolution cell, g(ϕ,S)≜IDFT(E) denotes the compact form of observation function, $f_{i}$ and S are known parameters, γ≈0, ϕ can be obtained through Equations (4) or (5) and n is the observation noise vector.

### 2.2. CBMeMBer Filter

The CBMeMBer filter is outlined as follows and the details can be found in [18,21]. It approximates the posterior multi-target density by a multi-Bernoulli RFS. The multi-Bernoulli RFS consists of M independent Bernoulli RFSs $X^{\left(i\right)}$, that is, $X=\cup_{i=1}^{M}X^{\left(i\right)}$. The probability density of Bernoulli RFS $X^{\left(i\right)}$ is

(11) $$\pi (X^{\left(i\right)})=\left\{ \begin{array}{l} 1-r^{\left(i\right)},X^{\left(i\right)}=\emptyset \\ r^{\left(i\right)}p^{\left(i\right)}(x),X^{\left(i\right)}=\{x\} \end{array}\right.$$

where $r^{\left(i\right)}\in [0,1]$ is the target existence probability and $p^{\left(i\right)}(\cdot )$ is a spatial distribution. Therefore, the probability density of multi-Bernoulli RFS X is given by

(12) $$\pi (X)=\prod_{i=1}^{M}(1-r^{\left(i\right)})\sum_{1\leq i_{1}\neq \cdots \neq i_{n}\leq M}^{}\prod_{j=1}^{n}\frac{r^{\left(i_{j}^{}\right)}p^{\left(i_{j}^{}\right)}(x_{j})}{1-r^{\left(i_{j}^{}\right)}}$$

where n is the number of targets.

The multi-Bernoulli RFS X is completely described by the multi-Bernoulli parameter set ${\left\{(r^{\left(i\right)},p^{\left(i\right)}(x))\right\}}_{i=1}^{M}$, and the probability density of the multi-Bernoulli RFSs X can be abbreviated by ${\left\{(r^{\left(i\right)},p^{\left(i\right)}(x))\right\}}_{i=1}^{M}$. The CBMeMBer filter consists of a prediction step and an update step.

#### 2.2.1. Prediction Step

If the posterior probability density at time k−1 is $\pi_{k-1}(X)={\left\{(r_{k-1}^{\left(i\right)},p_{k-1}^{\left(i\right)}(x_{k-1}))\right\}}_{i=1}^{M_{k-1}}$, then the predicted multi-target density is also a multi-Bernoulli formed by the union of the multi-Bernoulli for the surviving targets and target births

(13) $$\pi_{k|k-1}={\left\{\left(r_{P,k|k-1}^{\left(i\right)},p_{P,k|k-1}^{\left(i\right)}(x_{k})\right)\right\}}_{i=1}^{M_{k-1}}\cup {\left\{\left(r_{\Gamma ,k|k-1}^{\left(i\right)},p_{\Gamma ,k|k-1}^{\left(i\right)}(x_{k})\right)\right\}}_{i=1}^{M_{\Gamma ,k}}$$

where ${\left\{\left(r_{\Gamma ,k|k-1}^{\left(i\right)},p_{\Gamma ,k|k-1}^{\left(i\right)}(x_{k})\right)\right\}}_{i=1}^{M_{\Gamma ,k}}$ is the predicted multi-Bernoulli for the target births and it is usually assumed to be known. ${\left\{\left(r_{P,k|k-1}^{\left(i\right)},p_{P,k|k-1}^{\left(i\right)}(x_{k})\right)\right\}}_{i=1}^{M_{k-1}}$ is the predicted multi-Bernoulli for the surviving targets and it is given by

(14) $$r_{P,k|k-1}^{\left(i\right)}=r_{k-1}^{\left(i\right)}\langle p_{k-1}^{\left(i\right)}(x_{k-1}),p_{S,k}(x_{k-1})\rangle$$

(15) $$p_{P,k|k-1}^{\left(i\right)}(x_{k})=\frac{\langle f_{k|k-1}(x_{k}|x_{k-1}),p_{k-1}^{\left(i\right)}(x_{k-1})p_{S,k}(x_{k-1})\rangle }{\langle p_{k-1}^{\left(i\right)}(x_{k-1}),p_{S,k}(x_{k-1})\rangle }$$

where 〈f,g〉=∫f(x)g(x)dx denotes the inner product operation, $p_{S,k}(x_{k-1})$ is the survival probability of the surviving targets, $f_{k|k-1}(x_{k}|x_{k-1})$ is the single-target transition density. There are $M_{k|k-1}=M_{k-1}+M_{\Gamma ,k-1}$ predicted hypothesized tracks.

#### 2.2.2. Update Step

Assuming that $n_{k,z}$ measurements are collected as $Z_{k}=\{z_{k,1},\cdots ,z_{k,n_{k,z}}\}$ and the predicted probability density is $\pi_{k|k-1}(X)={\left\{(r_{k|k-1}^{\left(i\right)},p_{k|k-1}^{\left(i\right)}(x_{k}))\right\}}_{i=1}^{M_{k|k-1}}$, then the posterior multi-target density at time k can be approximated by a multi-Bernoulli as

(16) $$\pi_{k}\approx {\left\{\left(r_{L,k}^{\left(i\right)},p_{L,k}^{\left(i\right)}(x_{k})\right)\right\}}_{i=1}^{M_{k|k-1}}\cup {\left\{\left(r_{U,k}^{*}(z),p_{U,k}^{*}(x_{k};z)\right)\right\}}_{z\in Z_{k}}$$

The first term in Equation (16) corresponds to the multi-Bernoulli density for the legacy tracks and it can be given by

(17) $$r_{L,k}^{\left(i\right)}=r_{k|k-1}^{\left(i\right)}\frac{1-\langle p_{k|k-1}^{\left(i\right)}(x_{k}),p_{D,k}(x_{k})\rangle }{1-r_{k|k-1}^{\left(i\right)}\langle p_{k|k-1}^{\left(i\right)}(x_{k}),p_{D,k}(x_{k})\rangle }$$

(18) $$p_{L,k}^{\left(i\right)}(x_{k})=p_{k|k-1}^{\left(i\right)}(x_{k})\frac{1-p_{D,k}(x_{k})}{1-\langle p_{k|k-1}^{\left(i\right)}(x_{k}),p_{D,k}(x_{k})\rangle }$$

where $p_{D,k}(x_{k})$ is the detection probability.

The second term in Equation (16) corresponds to the multi-Bernoulli density for measurement-corrected tracks and it can be given as

(19) $$r_{U,k}^{*}(z)=\frac{\sum_{i=1}^{M_{k|k-1}}\frac{r_{k|k-1}^{\left(i\right)}(1-r_{k|k-1}^{\left(i\right)})\langle p_{k|k-1}^{\left(i\right)}(x_{k}),g_{k}(z|x_{k})p_{D,k}(x_{k})\rangle }{{\left(1-r_{k|k-1}^{\left(i\right)}\langle p_{k|k-1}^{\left(i\right)}(x_{k}),p_{D,k}(x_{k})\rangle \right)}^{2}}}{\kappa (z)+\sum_{i=1}^{M_{k|k-1}}\frac{r_{k|k-1}^{\left(i\right)}\langle p_{k|k-1}^{\left(i\right)}(x_{k}),g_{k}(z|x_{k})p_{D,k}(x_{k})\rangle }{1-r_{k|k-1}^{\left(i\right)}\langle p_{k|k-1}^{\left(i\right)}(x_{k}),p_{D,k}(x_{k})\rangle }}$$

(20) $$p_{U,k}^{*}(x_{k};z)=\frac{\sum_{i=1}^{M_{k|k-1}}\frac{r_{k|k-1}^{\left(i\right)}p_{k|k-1}^{\left(i\right)}(x_{k})p_{D,k}g_{k}(z|x_{k})}{1-r_{k|k-1}^{\left(i\right)}}}{\sum_{i=1}^{M_{k|k-1}}\frac{r_{k|k-1}^{\left(i\right)}}{1-r_{k|k-1}^{\left(i\right)}}\langle p_{k|k-1}^{\left(i\right)}(x_{k}),p_{D,k}(x_{k})g_{k}(z|x_{k})\rangle }$$

where $g_{k}(z|x_{k})$ is the likelihood function, κ(z) is the clutter intensity function. There are $M_{k}=M_{k|k-1}+n_{k,z}$ updated hypothesized tracks.

## 3. JTC Method Based on SCM and CBMeMBer Filter

In this section, the SCM-based JTC method is first presented by using the HRRP as the feature for target classification. Then, the SCM-JTC-CBMeMBer filter is derived for multi-target JTC.

### 3.1. SCM-Based JTC Method: Single-Target Case

The joint target state can be modeled as $\xi_{k-1}≜(x_{k-1},c)$, where $x_{k-1}$ is the kinematic state and c is the class label that can be taken from the set of the target classes $C=\{c^{1},c^{2},\cdots ,c^{n_{c}}\}$. $n_{c}$ and $c^{m}$ represent the total number of the target class and the *m*th target class, respectively. In the SCM-based JTC method, the available measurement at time k consists of kinematic (position) measurement $z_{k}^{p}={\left[r,\theta \right]}^{T}$ and signature (HRRP) measurement $z_{k}^{c}=d$, and the joint measurement is denoted as ${\tilde{z}}_{k}≜(z_{k}^{p},z_{k}^{c})$. The measurement set up to time k is represented by ${\tilde{Z}}^{k}={\left\{{\tilde{z}}_{\tau }\right\}}_{\tau =0}^{k}$.

The purpose of Bayesian JTC is to estimate the target state and class simultaneously at time k, under the condition that the distribution $p(x_{k-1},c|{\tilde{Z}}^{k-1})$ at time k−1 and the measurement ${\tilde{z}}_{k}$ at time k are available. That is, to obtain the posterior probability-mass distribution

(21) $$p(x_{k},c|{\tilde{Z}}^{k})=p(x_{k}|c,{\tilde{Z}}^{k})p(c|{\tilde{Z}}^{k})$$

For target tracking, the class-dependent probability density function (PDF) for a specific target class $c^{m}$ can be represented as

(22) $$p(x_{k}|c^{m},{\tilde{Z}}^{k})=\frac{p({\tilde{Z}}_{k}|x_{k},c^{m})p(x_{k}|c^{m},{\tilde{Z}}^{k-1})}{p({\tilde{z}}_{k}|c^{m},{\tilde{Z}}^{k-1})}$$

where $p({\tilde{z}}_{k}|c^{m},{\tilde{Z}}^{k-1})=\int p({\tilde{z}}_{k}|x_{k},c^{m})p(x_{k}|c^{m},{\tilde{Z}}^{k-1})dx_{k}$ is the normalized factor. 

Accordingly, for target classification, the probability function can be obtained by

(23) $$\mu_{k}^{m}≜p(c^{m}|{\tilde{Z}}^{k})=\frac{p({\tilde{z}}_{k}|c^{m},{\tilde{Z}}^{k-1})p(c^{m}|{\tilde{Z}}^{k-1})}{p({\tilde{z}}_{k}|{\tilde{Z}}^{k-1})}$$

where $p({\tilde{z}}_{k}|{\tilde{Z}}^{k-1})=\sum_{m=1}^{n_{c}}p({\tilde{z}}_{k}|c^{m},{\tilde{Z}}^{k-1})p(c^{m}|{\tilde{Z}}^{k-1})$ is the normalized factor.

To obtain the recursive equations of the SCM-based JTC method, two assumptions should be followed.

**Assumption** **1.** *All the targets have the same motion model, i.e., the single state transition function* $f_{k|k-1}(x_{k},c^{j}|x_{k-1},c^{m})$ *is*

(24) $$f_{k|k-1}(x_{k},c^{j}|x_{k-1},c^{m})=f_{k|k-1}^{k}(x_{k}|x_{k-1})f_{k|k-1}^{c}(c^{j}|c^{m})$$

*where* $f_{k|k-1}^{k}(x_{k}|x_{k-1})$ *is the kinematic state transition function and is decided by the system model and* $f_{k|k-1}^{c}(c^{j}|c^{m})$ *is the class state transition function and can be represented by the Dirac function* δ(⋅) *as*

(25) $$f_{k|k-1}^{c}(c^{j}|c^{m})=\delta_{m}(j)=\left\{ \begin{array}{l} 1,\mathrm{if}\;j=m\\ 0,\mathrm{if}\;j\neq m \end{array}\right.$$

**Assumption** **2.** *The kinematic measurement and HRRP measurement are independent of each other, and the kinematic measurement is independent of the target class, so the measurement likelihood can be written as*

(26) $$p({\tilde{z}}_{k}|x_{k},c^{m},{\tilde{Z}}^{k-1})=p(z_{k}^{p}|x_{k})p(z_{k}^{c}|x_{k},c^{m})$$

*where* $p(z_{k}^{p}|x_{k})≜g_{k}^{k}(x_{k})=N(z_{k};h(x_{k}),R)$ *is the likelihood function of kinematic measurement.* $p(z_{k}^{c}|x_{k},c^{m})≜g_{k}^{c}(x_{k},c^{m})=\langle d_{k},g(\phi_{k},S_{c^{m}})\rangle /\left(\left\|d_{k}\right\|\cdot \left\|g(\phi_{k},S_{c^{m}})\right\|\right)$ *is the likelihood function of HRRP measurement, and is defined as normalized correlation coefficient of observed HRRP with model-predicted HRRP.* $S_{c^{m}}$ *is the SCM corresponding to target class* $c^{m}$ *.*

Therefore, the SCM-based JTC method can be constructed through the following two steps.

The prediction steps of the target state and class are given by

(27) $$p(x_{k}|c^{m},{\tilde{Z}}^{k-1})=\int f_{k|k-1}^{k}(x_{k}|x_{k-1})p(x_{k-1}|c^{m},{\tilde{Z}}^{k-1})dx_{k-1}$$

(28) $$\mu_{k|k-1}^{m}=\mu_{k-1}^{m}$$

Similarly, the update steps of target state and class are

(29) $$p(x_{k}|c^{m},{\tilde{Z}}^{k})=\frac{g_{k}^{k}(x_{k})g_{k}^{c}(x_{k},c^{m})p(x_{k}|c^{m},{\tilde{Z}}^{k-1})}{p({\tilde{z}}_{k}|c^{m},{\tilde{Z}}^{k-1})}$$

(30) $$\mu_{k}^{m}=p(c^{m}|{\tilde{Z}}^{k})=\frac{p({\tilde{z}}_{k}|c^{m},{\tilde{Z}}^{k-1})p(c^{m}|{\tilde{Z}}^{k-1})}{p({\tilde{z}}_{k}|{\tilde{Z}}^{k-1})}$$

with

(31) $$p({\tilde{z}}_{k}|c^{m},Z^{k-1})=\int g_{k}^{k}(x_{k})g_{k}^{c}(x_{k},c_{}^{m})p(x_{k}|c_{}^{m},Z^{k-1})dx_{k}$$

(32) $$p({\tilde{z}}_{k}|{\tilde{Z}}^{k-1})=\sum_{m=1}^{n_{c}}p({\tilde{z}}_{k}|c_{}^{m},{\tilde{Z}}^{k-1})p(c_{}^{m}|{\tilde{Z}}^{k-1})$$

Because of the complexity and high nonlinearity of the observation model, there is no analytic form to obtain the recursive estimation of target state and class, so we have to resort to the SMC technique (also known as particle filter, PF). Given the particle set ${\left\{w_{k-1}^{j},x_{k-1}^{j},l^{j}\right\}}_{j=1}^{n_{k-1,p}}$, where the superscript j denotes the index of the particles, $l^{j}\in C$ represents the class label corresponding to the *j*th particle and $n_{k-1,p}$ is the number of particles at time k−1. The posterior target state and associated class probability can be represented by

(33) $$p(x_{k-1}|c_{}^{m},{\tilde{Z}}^{k-1})=\sum_{j=1}^{n_{k-1,p}}w_{k-1}^{m,j}\delta_{x_{k-1}^{m,j}}(x_{k-1})$$

(34) $$p(c^{m}|Z^{k-1})=\sum_{j=1}^{n_{k-1,p}^{}}w_{k-1}^{j}\delta_{l^{j}}(c^{m})/\sum_{j=1}^{n_{k-1,p}}w_{k-1}^{j}$$

with

(35) $$w_{k-1}^{m,j}=w_{k-1}^{j}\delta_{l^{j}}(c^{m})/\sum_{j=1}^{n_{k-1,p}}w_{k-1}^{j}\delta_{l^{j}}(c^{m})$$

(36) $$x_{k-1}^{m,j}=x_{k-1}^{j}\delta_{l^{j}}(c^{m})$$

Then, a complete recursive procedure from time k−1 to k can be summarized as Algorithm 1.
**Algorithm 1** Single-time step recursion of the scattering center model (SCM)-based joint tracking and classification (JTC) methodStep 1.Model prediction1)Target state prediction: $x_{k}^{j}=Fx_{k-1}^{j}+w_{k-1}^{j}$2)Kinematic observation prediction: ${\hat{z}}_{k}^{j}=h(x_{k}^{j})$3)Aspect angle prediction: $\phi_{k}^{j}=\cos^{-1}\left(\frac{x_{k}^{j}{\dot{x}}_{k}^{j}+y_{k}^{j}{\dot{y}}_{k}^{j}}{\sqrt{{\left(x_{k}^{j}\right)}^{2}+{\left(y_{k}^{j}\right)}^{2}}\sqrt{{\left({\dot{x}}_{k}^{j}\right)}^{2}+{\left({\dot{y}}_{k}^{j}\right)}^{2}}}\right)$4)HRRP prediction: ${\hat{d}}_{k}^{j}=g(\phi_{k}^{j},S_{l^{j}})$where $S_{l^{j}}$ represents the 3D-SCM corresponding to target class $l^{j}$.Step 2.Likelihood evaluation
1)Kinematic observation likelihood: $g_{k}^{k}(x_{k}^{j})=N(z_{k};h(x_{k}^{j}),R)$2)HRRP correlation coefficient: $g_{k}^{c}(x_{k}^{j},l^{j})=\langle d_{k},{\hat{d}}_{k}^{j}\rangle /\left(\left\|d_{k}\right\|\cdot \left\|{\hat{d}}_{k}^{j}\right\|\right)$Step 3.Particle weight evaluation1)Joint weight calculation: $w_{k}^{j}=w_{k-1}^{j}\cdot g_{k}^{k}(x_{k}^{j})\cdot g_{k}^{c}(x_{k}^{j},l^{j})$2)Normalization of weights: ${\tilde{w}}_{k}^{j}=w_{k}^{j}/\sum_{j=1}^{n_{k-1,p}}w_{k}^{j}$

The posterior target state estimation ${\hat{x}}_{k}$ and class probabilities $p(c^{m}|{\tilde{Z}}^{k})$ at time k can be obtained by

(37) $${\hat{x}}_{k}=\sum_{m=1}^{n_{c}}p(c^{m}|{\tilde{Z}}^{k}){\hat{x}}_{k}^{m}$$

(38) $$p(c^{m}|{\tilde{Z}}^{k})=\sum_{j=1}^{n_{k-1,p}}{\tilde{w}}_{k}^{j}\delta_{l^{j}}(c^{m})/\sum_{j=1}^{n_{k-1,p}}{\tilde{w}}_{k}^{j}$$

with

(39) $${\hat{x}}_{k}^{m}=\sum_{j=1}^{n_{k-1,p}}{\tilde{w}}_{k}^{m,j}\delta_{x_{k}^{m,j}}(x_{k})$$

(40) $${\tilde{w}}_{k}^{m,j}={\tilde{w}}_{k}^{j}\delta_{l^{j}}(c^{m})/\sum_{j=1}^{n_{k-1,p}}{\tilde{w}}_{k}^{j}\delta_{l^{j}}(c^{m})$$

(41) $$x_{k}^{m,j}=x_{k}^{j}\delta_{l^{j}}(c^{m})$$

To reduce the effect of particle degeneracy, the resampling operation [33] should be considered in method implementation. Specifically, the class-dependent resampling strategy is adopted to avoid particle degeneracy caused by an incorrect target classification. In this strategy, the maximum number of particles for the SCM-based JTC method is set as $L_{\max}$, while the minimum number of particles for each target class is set as $L_{\min}$. In this paper, the standard resampling operation is used to resample each class, that is, for the particle with $l^{j}=c^{m}$, ${\left\{x_{k}^{j^{\prime }},1/n_{k,p,m},l^{j^{\prime }}\right\}}_{j^{\prime }=1}^{n_{k,p,m}}=\mathrm{resample}{\left\{x_{k}^{j},{\tilde{w}}_{k}^{j},l^{j}\right\}}_{l^{j}=c^{m}}$, $n_{k,p,m}≜\max(L_{\min},n_{k-1,p}\cdot p(c^{m}|{\tilde{Z}}^{k}))$.

### 3.2. SCM-JTC-CBMeMBer Filter: Multi-Target Case

For the JTC of multi-target, the available measurement set at time k is denoted as $Z_{k}={\left\{{\tilde{z}}_{k,l}\right\}}_{l=0}^{n_{k,z}}$ and the measurement set up to time k is $Z^{k}={\left\{Z_{l}\right\}}_{l=1}^{k}$. The posterior multi-target density at time k−1 is modeled as a multi-Bernoulli

(42) $$\begin{array}{ll} \pi_{k-1} & ={\left\{\left(r_{k-1}^{\left(i\right)},p_{k-1}^{\left(i\right)}(x_{k-1}|Z^{k-1})\right)\right\}}_{i=1}^{M_{k-1}}\\ & ={\left\{\left(r_{k-1}^{\left(i\right)},\sum_{m=1}^{n_{c}}p_{k-1}^{\left(i\right)}(x_{k-1}|c^{m},Z^{k-1})p_{k-1}^{\left(i\right)}(c^{m}|Z^{k-1})\right)\right\}}_{i=1}^{M_{k-1}} \end{array}$$

In addition to Assumption 1 and Assumption 2, the following assumptions should also be followed to obtain the SCM-JTC-CBMeMBer filter.

**Assumption** **3.** *Each target evolves motion and generates measurements independently.*

**Assumption** **4.** *The clutter is modeled as Poisson RFS with Poisson average rate* $\lambda_{c}$ *, and it is independent of target-originated measurements. The spatial distribution of the clutter is a uniform distribution, denoted by* $C(\tilde{z})$ *. The clutter intensity function is* $\kappa (\tilde{z})=\lambda_{c}C(\tilde{z})$ *.*

**Assumption** **5.** *The survival and detection probabilities are state-independent, i.e.,* $p_{S,k}(x,c)=p_{S,k}$ *,* $p_{D,k}(x,c)=p_{D,k}$ *.*

**Assumption** **6.** *The PDF of birth targets at time* k−1 *is also a multi-Bernoulli, namely*

(43) $$\begin{array}{ll} \pi_{B,k-1} & ={\left\{\left(r_{B,k-1}^{\left(i\right)},p_{B,k-1}^{\left(i\right)}(x_{k-1}|Z^{k-1})\right)\right\}}_{i=1}^{M_{B,k-1}}\\ & ={\left\{\left(r_{B,k-1}^{\left(i\right)},\sum_{m=1}^{n_{c}}p_{B,k-1}^{\left(i\right)}(x_{k-1}|c^{m},Z^{k-1})p_{B,k-1}^{\left(i\right)}(c^{m}|Z^{k-1})\right)\right\}}_{i=1}^{M_{B,k-1}} \end{array}$$

**Proposition** **1.** *If the posterior multi-target density at time* k−1 *is a multi-Bernoulli, as shown in Equation (42), then the predicted multi-target density is also a multi-Bernoulli and is given by*

(44) $$\begin{array}{c} \pi_{k|k-1}={\left\{\left(r_{P,k|k-1}^{\left(i\right)},p_{P,k|k-1}^{\left(i\right)}(x_{k}|Z^{k-1})\right)\right\}}_{i=1}^{M_{k-1}}\cup {\left\{\left(r_{\Gamma ,k|k-1}^{\left(i\right)},p_{\Gamma ,k|k-1}^{\left(i\right)}(x_{k}|Z^{k-1})\right)\right\}}_{i=1}^{M_{\Gamma ,k}}\\ ={\left\{\left(r_{P,k|k-1}^{\left(i\right)},\sum_{m=1}^{n_{c}}p_{P,k|k-1}^{\left(i\right)}(x_{k}|c^{m},Z^{k-1})p_{P,k|k-1}^{\left(i\right)}(c^{m}|Z^{k-1})\right)\right\}}_{i=1}^{M_{k-1}}\cup {\left\{\left(r_{\Gamma ,k|k-1}^{\left(i\right)},p_{\Gamma ,k|k-1}^{\left(i\right)}(x_{k}|Z^{k-1})\right)\right\}}_{i=1}^{M_{\Gamma ,k}} \end{array}$$

*with*

(45) $$r_{P,k|k-1}^{\left(i\right)}=r_{k-1}^{\left(i\right)}\sum_{m=1}^{n_{c}}p_{k-1}^{\left(i\right)}(c^{m}|Z^{k-1})\langle p_{k-1}^{\left(i\right)}(x_{k-1}|c^{m},Z^{k-1}),p_{S,k}\rangle$$

(46) $$p_{P,k|k-1}^{\left(i\right)}(c^{m}|Z^{k-1})=p_{k-1}^{\left(i\right)}(c^{m}|Z^{k-1})$$

(47) $$p_{P,k|k-1}^{\left(i\right)}(x_{k}|c^{m},Z^{k-1})=\frac{\langle f_{k|k-1}^{k}(x_{k}|x_{k-1}),p_{k-1}^{\left(i\right)}(x_{k-1}|c^{m},Z^{k-1})p_{S,k}\rangle }{\sum_{m=1}^{n_{c}}p_{k-1}^{\left(i\right)}(c^{m}|Z^{k-1})\langle p_{k-1}^{\left(i\right)}(x_{k-1}|c^{m},Z^{k-1}),p_{S,k}\rangle }$$

(48) $$r_{\Gamma ,k|k-1}^{\left(i\right)}=r_{B,k-1}^{\left(i\right)}$$

(49) $$p_{\Gamma ,k|k-1}^{\left(i\right)}(x_{k}|Z^{k-1})=\sum_{m=1}^{n_{c}}p_{B,k-1}^{\left(i\right)}(x_{k-1}|c^{m},Z^{k-1})p_{B,k-1}^{\left(i\right)}(c^{m}|Z^{k-1})$$

The proof of Proposition 1 is given in Appendix A.

**Proposition** **2.** *If the predicted multi-target density at time k is a multi-Bernoulli*

(50) $$\begin{array}{ll} \pi_{k|k-1} & ={\left\{\left(r_{k|k-1}^{\left(i\right)},p_{k|k-1}^{\left(i\right)}(x_{k}|Z^{k-1})\right)\right\}}_{i=1}^{M_{k|k-1}}\\ & ={\left\{\left(r_{k|k-1}^{\left(i\right)},\sum_{m=1}^{n_{c}}p_{k|k-1}^{\left(i\right)}(x_{k}|c^{m},Z^{k-1})p_{k|k-1}^{\left(i\right)}(c^{m}|Z^{k-1})\right)\right\}}_{i=1}^{M_{k|k-1}} \end{array}$$

Then, the posterior multi-target density can be approximated by a multi-Bernoulli as

(51) $$\begin{array}{ll} \pi_{k} & \approx {\left\{\left(r_{L,k}^{\left(i\right)},p_{L,k}^{\left(i\right)}(x_{k}|Z^{k})\right)\right\}}_{i=1}^{M_{k|k-1}}\cup {\left\{\left(r_{U,k}^{*}(\tilde{z}),p_{U,k}^{*}(\tilde{z})\right)\right\}}_{\tilde{z}\in Z_{k}}\\ & ={\left\{\left(r_{L,k}^{\left(i\right)},\sum_{m=1}^{n_{c}}p_{L,k}^{\left(i\right)}(x_{k}|c^{m},Z^{k})p_{L,k}^{\left(i\right)}(c^{m}|Z^{k})\right)\right\}}_{i=1}^{M_{k|k-1}}\cup {\left\{\left(r_{U,k}^{*}(\tilde{z}),\sum_{m=1}^{n_{c}}p_{U,k}^{\left(i\right)}(x_{k}|c^{m},Z^{k})p_{U,k}^{\left(i\right)}(c^{m}|Z^{k})\right)\right\}}_{\tilde{z}\in Z_{k}} \end{array}$$

with

(52) $$r_{L,k}^{\left(i\right)}=r_{k|k-1}^{\left(i\right)}\frac{1-\sum_{m=1}^{n_{c}}p_{k|k-1}^{\left(i\right)}(c^{m}|Z^{k-1})\langle p_{k|k-1}^{\left(i\right)}(x_{k}|c^{m},Z^{k-1}),p_{D,k}\rangle }{1-r_{k|k-1}^{\left(i\right)}\sum_{m=1}^{n_{c}}p_{k|k-1}^{\left(i\right)}(c^{m}|Z^{k-1})\langle p_{k|k-1}^{\left(i\right)}(x_{k}|c^{m},Z^{k-1}),p_{D,k}\rangle }$$

(53) $$p_{L,k}^{\left(i\right)}(x_{k}|c^{m},Z^{k})=\frac{\left(1-p_{D,k})p_{k|k-1}^{\left(i\right)}(x_{k}|c^{m},Z^{k-1}\right)}{1-\sum_{m=1}^{n_{c}}p_{k|k-1}^{\left(i\right)}(c^{m}|Z^{k-1})\langle p_{k|k-1}^{\left(i\right)}(x_{k}|c^{m},Z^{k-1}),p_{D,k}\rangle }$$

(54) $$p_{L,k}^{\left(i\right)}(c^{m}|Z^{k})=p_{k|k-1}^{\left(i\right)}(c^{m}|Z^{k-1})$$

(55) $$r_{U,k}^{*}(\tilde{z})=\frac{\sum_{i=1}^{M_{k|k-1}}\frac{r_{k|k-1}^{\left(i\right)}(1-r_{k|k-1}^{\left(i\right)})\sum_{m=1}^{n_{c}}p_{k|k-1}^{\left(i\right)}(c^{m}|Z^{k-1})\langle p_{k|k-1}^{\left(i\right)}(x_{k}|c^{m},Z^{k-1}),g_{k}^{k}(x_{k})g_{k}^{c}(x_{k},c^{m})p_{D,k}\rangle }{{\left(1-r_{k|k-1}^{\left(i\right)}\sum_{m=1}^{n_{c}}p_{k|k-1}^{\left(i\right)}(c^{m}|Z^{k-1})\langle p_{k|k-1}^{\left(i\right)}(x_{k}|c^{m},Z^{k-1}),p_{D,k}\rangle \right)}^{2}}}{\kappa (\tilde{z})+\sum_{i=1}^{M_{k|k-1}}\frac{r_{k|k-1}^{\left(i\right)}\sum_{m=1}^{n_{c}}p_{k|k-1}^{\left(i\right)}(c^{m}|Z^{k-1})\langle p_{k|k-1}^{\left(i\right)}(x_{k}|c^{m},Z^{k-1}),g_{k}^{k}(x_{k})g_{k}^{c}(x_{k},c^{m})p_{D,k}\rangle }{1-r_{k|k-1}^{\left(i\right)}\sum_{m=1}^{n_{c}}p_{k|k-1}^{\left(i\right)}(c^{m}|Z^{k-1})\langle p_{k|k-1}^{\left(i\right)}(x_{k}|c^{m},Z^{k-1}),p_{D,k}\rangle }}$$

(56) $$p_{U,k}^{\left(i\right)}(x_{k}|c^{m},Z^{k})=\frac{\sum_{i=1}^{M_{k|k-1}}\frac{r_{k|k-1}^{\left(i\right)}p_{k|k-1}^{\left(i\right)}(x_{k}|c^{m},Z^{k-1})p_{k|k-1}^{\left(i\right)}(c^{m}|Z^{k-1})p_{D,k}g_{k}^{k}(x_{k})g_{k}^{c}(x_{k},c_{}^{m})}{1-r_{k|k-1}^{\left(i\right)}}}{\sum_{i=1}^{M_{k|k-1}}\frac{r_{k|k-1}^{\left(i\right)}p_{k|k-1}^{\left(i\right)}(c^{m}|Z^{k-1})\langle p_{k|k-1}^{\left(i\right)}(x_{k}|c^{m},Z^{k-1}),p_{D,k}g_{k}^{k}(x_{k})g_{k}^{c}(x_{k},c^{m})\rangle }{1-r_{k|k-1}^{\left(i\right)}}}$$

(57) $$p_{U,k}^{\left(i\right)}(c^{m}|Z^{k})=\frac{\sum_{i=1}^{M_{k|k-1}}\frac{r_{k|k-1}^{\left(i\right)}p_{k|k-1}^{\left(i\right)}(c^{m}|Z^{k-1})\langle p_{k|k-1}^{\left(i\right)}(x_{k}|c^{m},Z^{k-1}),p_{D,k}g_{k}^{k}(x_{k})g_{k}^{c}(x_{k},c_{}^{m})\rangle }{1-r_{k|k-1}^{\left(i\right)}}}{\sum_{i=1}^{M_{k|k-1}}\frac{r_{k|k-1}^{\left(i\right)}}{1-r_{k|k-1}^{\left(i\right)}}\sum_{m=1}^{n_{c}}p_{k|k-1}^{\left(i\right)}(c^{m}|Z^{k-1})\langle p_{k|k-1}^{\left(i\right)}(x_{k}|c^{m},Z^{k-1}),p_{D,k}g_{k}^{k}(x_{k})g_{k}^{c}(x_{k},c_{}^{m})\rangle }$$

The proof of Proposition 2 is shown in Appendix B.

The state extraction step is similar to the CBMeMBer filter, and the details can be found in [22]. 

## 4. SMC Implementation of the SCM-JTC-CBMeMBer Filter

In what follows, SMC implementation of the SCM-JTC-CBMeMBer filter recursion will be presented.

Supposing that the posterior multi-target density $\pi_{k-1}={\left\{\left(r_{k-1}^{\left(i\right)},p_{k-1}^{\left(i\right)}(x_{k-1}|Z^{k-1})\right)\right\}}_{i=1}^{M_{k-1}}$ is given, and each component $p_{k-1}^{\left(i\right)}(x_{k-1}|Z^{k-1})$ is comprised of $n_{k-1}^{i}$ weighted particles ${\left\{w_{k-1}^{i,j},x_{k-1}^{i,j},l^{i,j}\right\}}_{j=1}^{n_{k-1}^{i}}$, that is

(58) $$p_{k-1}^{\left(i\right)}(x_{k-1}|Z^{k-1})=\sum_{j=1}^{n_{k-1}^{i}}w_{k-1}^{i,j}\delta_{x_{k-1}^{i,j}}(x_{k-1})$$

Then the class probability $p_{k-1}^{\left(i\right)}(c^{m}|Z^{k-1})$ and the kinematic state distribution conditioned on classification $p_{k-1}^{\left(i\right)}(x_{k-1}|c^{m},Z^{k-1})$ can be obtained by

(59) $$p_{k-1}^{\left(i\right)}(c^{m}|Z^{k-1})=\sum_{j=1}^{n_{k-1}^{i}}w_{k-1}^{i,j}\delta_{l^{i,j}}(c^{m})/\sum_{j=1}^{n_{k-1}^{i}}w_{k-1}^{i,j}$$

(60) $$p_{k-1}^{\left(i\right)}(x_{k-1}|c^{m},Z^{k-1})=\sum_{j=1}^{n_{k-1}^{i}}w_{k-1}^{i,m,j}\delta_{x_{k-1}^{i,m,j}}(x_{k-1})$$

with

(61) $$w_{k-1}^{i,m,j}=w_{k-1}^{i,j}\delta_{l^{i,j}}(c^{m})/\sum_{j=1}^{n_{k-1}^{i}}w_{k-1}^{i,j}\delta_{l^{i,j}}(c^{m})$$

(62) $$x_{k-1}^{i,m,j}=x_{k-1}^{i,j}\delta_{l^{i,j}}(c^{m})$$

**Proposition** **3.** *Given the importance density* $q_{k}^{\left(i\right)}(\cdot |x_{k-1},Z_{k})$ *of the posterior distribution and importance densities* $b_{k}^{\left(i\right)}(\cdot |x_{k-1},Z_{k})$ *of the birth targets, according to Proposition 1, if the prior distribution is multi-Bernoulli* $\pi_{k-1}={\left\{\left(r_{k-1}^{\left(i\right)},\sum_{m=1}^{n_{c}}p_{k-1}^{\left(i\right)}(x_{k-1}|c^{m},Z^{k-1})p_{k-1}^{\left(i\right)}(c^{m}|Z^{k-1})\right)\right\}}_{i=1}^{M_{k-1}}$ *and each* $p_{k-1}^{\left(i\right)}=\sum_{m=1}^{n_{c}}p_{k-1}^{\left(i\right)}(x_{k-1}|c^{m},Z^{k-1})p_{k-1}^{\left(i\right)}(c^{m}|Z^{k-1})$ *is comprised of* $n_{k-1}^{i}$ *weighted particles* ${\left\{w_{k-1}^{i,j},x_{k-1}^{i,j},l_{}^{i,j}\right\}}_{j=1}^{n_{k-1}^{i}}$ *, then the predicted multi-target density is also multi-Bernoulli and the SMC implementation is calculated as*

(63) $$r_{P,k|k-1}^{\left(i\right)}=r_{k-1}^{\left(i\right)}\sum_{j=1}^{n_{k-1}^{i}}w_{k-1}^{i,j}p_{S,k}$$

(64) $$p_{P,k|k-1}^{\left(i\right)}(c^{m}|Z^{k-1})=\sum_{j=1}^{n_{k-1}^{i}}w_{P,k|k-1}^{i,j}\delta_{l^{i,j}}(c^{m})/\sum_{j=1}^{n_{k-1}^{i}}w_{P,k|k-1}^{i,j}$$

(65) $$p_{P,k|k-1}^{\left(i\right)}(x_{k}|c^{m},Z^{k-1})=\sum_{j=1}^{n_{k-1}^{i}}w_{P,k-1}^{i,m,j}\delta_{x_{P,k|k-1}^{i,m,j}}(x_{k})$$

(66) $$r_{\Gamma ,k|k-1}^{\left(i\right)}=r_{B,k-1}^{\left(i\right)}$$

(67) $$p_{\Gamma ,k|k-1}^{\left(i\right)}(x_{k}|c^{m},Z^{k-1})=\sum_{j=1}^{n_{k-1}^{i}}w_{B,k|k-1}^{i,m,j}\delta_{x_{B,k|k-1}^{i,m,j}}(x_{k})$$

(68) $$p_{\Gamma ,k|k-1}^{\left(i\right)}(c^{m}|Z^{k-1})=\sum_{j=1}^{n_{k-1}^{i}}w_{B,k|k-1}^{i,j}\delta_{l^{i,j}}(c^{m})/\sum_{j=1}^{n_{k-1}^{i}}w_{B,k|k-1}^{i,j}$$

*with*

(69) $$x_{P,k|k-1}^{i,j}∼q_{k}^{\left(i\right)}(\cdot |x_{k-1},Z_{k})$$

(70) $$x_{B,k|k-1}^{i,j}∼b_{k}^{\left(i\right)}(\cdot |x_{k-1},Z_{k})$$

(71) $${\tilde{w}}_{P,k|k-1}^{i,j}=w_{k-1}^{i,j}p_{S,k}$$

(72) $$w_{P,k-1}^{i,j}={\tilde{w}}_{P,k|k-1}^{i,j}/\sum_{j=1}^{n_{k-1}^{i}}{\tilde{w}}_{P,k|k-1}^{i,j}$$

(73) $$w_{P,k|k-1}^{i,m,j}=w_{P,k|k-1}^{i,j}\delta_{l^{i,j}}(c^{m})/\sum_{j=1}^{n_{k-1}^{i}}w_{P,k|k-1}^{i,j}\delta_{l^{i,j}}(c^{m})$$

(74) $$x_{P,k|k-1}^{i,m,j}=x_{P,k|k-1}^{i,j}\delta_{l^{i,j}}(c^{m})$$

(75) $$w_{B,k|k-1}^{i,j}=w_{B,k-1}^{i,j}$$

(76) $$w_{B,k|k-1}^{i,m,j}=w_{B,k|k-1}^{i,j}\delta_{l^{i,j}}(c^{m})/\sum_{j=1}^{n_{k-1}^{i}}w_{B,k|k-1}^{i,j}\delta_{l^{i,j}}(c^{m})$$

(77) $$x_{B,k|k-1}^{i,m,j}=x_{B,k|k-1}^{i,j}\delta_{l^{i,j}}(c^{m})$$

The proof of Proposition 3 is given in Appendix C.

**Proposition** **4.** *If the predicted multi-target density is the multi-Bernoulli* $\pi_{k|k-1}={\left\{\left(r_{k|k-1}^{\left(i\right)},\sum_{m=1}^{n_{c}}p_{k|k-1}^{\left(i\right)}(x_{k}|c^{m},Z^{k-1})p_{k|k-1}^{\left(i\right)}(c^{m}|Z^{k-1})\right)\right\}}_{i=1}^{M_{k|k-1}}$ *, then the updated multi-target density is also a multi-Bernoulli, and the SMC implementation can be computed by*

(78) $$r_{L,k}^{\left(i\right)}=r_{k|k-1}^{\left(i\right)}\frac{1-\rho_{L,k}^{i}}{1-r_{k|k-1}^{\left(i\right)}\rho_{L,k}^{i}}$$

(79) $$p_{L,k}^{\left(i\right)}(c^{m}|Z^{k})=\sum_{j=1}^{n_{k-1}^{i}}w_{k|k-1}^{i,j}\delta_{l^{i,j}}(c^{m})/\sum_{j=1}^{n_{k-1}^{i}}w_{k|k-1}^{i,j}$$

(80) $$p_{L,k}^{\left(i\right)}(x_{k}|c^{m},Z^{k})=\sum_{j=1}^{n_{k-1}^{i}}w_{L,k}^{i,m,j}\delta_{x_{k|k-1}^{i,m,j}}(x_{k})$$

(81) $$r_{U,k}^{*}(\tilde{z})=\frac{\sum_{i=1}^{M_{k|k-1}}\frac{r_{k|k-1}^{\left(i\right)}(1-r_{k|k-1}^{\left(i\right)})\rho_{U,k}^{i}}{{\left(1-r_{k|k-1}^{\left(i\right)}\rho_{L,k}^{i}\right)}^{2}}}{\kappa (\tilde{z})+\sum_{i=1}^{M_{k|k-1}}\frac{r_{k|k-1}^{\left(i\right)}\rho_{U,k}^{i}}{1-r_{k|k-1}^{\left(i\right)}\rho_{L,k}^{i}}}$$

(82) $$p_{U,k}^{\left(i\right)}(x_{k}|c^{m},Z^{k})=\sum_{i=1}^{M_{k|k-1}}\sum_{j=1}^{n_{k-1}^{i}}w_{U,k}^{i,m,j}\delta_{x_{k|k-1}^{i,m,j}}(x_{k})$$

(83) $$p_{U,k}^{\left(i\right)}(c^{m}|Z^{k})=\sum_{i=1}^{M_{k|k-1}}\sum_{j=1}^{n_{k-1}^{i}}w_{U,k}^{i,j}\delta_{l^{i,j}}(c^{m})/\sum_{i=1}^{M_{k|k-1}}\sum_{j=1}^{n_{k-1}^{i}}w_{U,k}^{i,j}$$

*with*

(84) $$\rho_{L,k}^{i}=\sum_{j=1}^{n_{k-1}^{i}}w_{k|k-1}^{i,j}p_{D,k}$$

(85) $$\rho_{U,k}^{i}=\sum_{j=1}^{n_{k-1}^{i}}w_{k|k-1}^{i,j}g_{k}^{k}(x_{k})g_{k}^{c}(x_{k},c_{}^{m})p_{D,k}$$

(86) $${\tilde{w}}_{L,k}^{i,j}=(1-p_{D,k})w_{k|k-1}^{i,j}$$

(87) $$w_{L,k}^{i,j}={\tilde{w}}_{L,k}^{i,j}/\sum_{j=1}^{n_{k-1}^{i}}{\tilde{w}}_{L,k}^{i,j}$$

(88) $$w_{L,k}^{i,m,j}=w_{L,k}^{i,j}\delta_{l^{i,j}}(c^{m})/\sum_{j=1}^{n_{k-1}^{i}}w_{L,k}^{i,j}\delta_{l^{i,j}}(c^{m})$$

(89) $$x_{L,k}^{i,m,j}=x_{L,k}^{i,j}\delta_{l^{i,j}}(c^{m})$$

(90) $${\tilde{w}}_{U,k}^{i,j}=\frac{r_{k|k-1}^{\left(i\right)}}{1-r_{k|k-1}^{\left(i\right)}}p_{D,k}w_{k|k-1}^{i,j}g_{k}^{k}(x_{k})g_{k}^{c}(x_{k},c_{}^{m})$$

(91) $$w_{U,k}^{i,j}={\tilde{w}}_{U,k}^{i,j}/\sum_{j=1}^{n_{k-1}^{i}}{\tilde{w}}_{U,k}^{i,j}$$

(92) $$w_{U,k}^{i,m,j}=\frac{w_{U,k}^{i,j}\delta_{l^{i,j}}(c^{m})}{\sum_{i=1}^{M_{k|k-1}}\sum_{j=1}^{n_{k-1}^{i}}w_{U,k}^{i,j}\delta_{l^{i,j}}(c^{m})}$$

The proof of Proposition 4 is detailed in Appendix D.

Particle resampling is also needed for SMC implementation of the SCM-JTC-CBMeMBer filter, and the same resampling strategy as introduced in Section 3.1 is used. Similar to the CBMeMBer filter, in the proposed SCM-JTC-CBMeMBer filter, the pruning and merge strategy (refer to [22] for the details) should be adopted to reduce the computational burden.

## 5. Simulation Results

**Figure 2:**
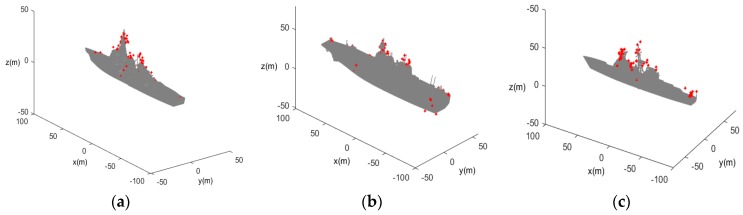
Target CAD models and the corresponding 3D scattering center models (3D-SCMs): (**a**) Target Class A; (**b**) Target Class B; (**c**) Target Class C.

The effectiveness of the proposed SCM-based JTC method and SMC-JTC-CBMeMBer filter is evaluated by simulations. The observation precisions of range and bearing are $\sigma_{r}=1\;m$ and $\sigma_{\beta }=0.3{}^{\circ}$, respectively. The radar is located at the origin of the coordinates with center frequency $f_{c}=35\;\mathrm{GHz}$, bandwidth B=150 MHz, frequency interval ΔF=1 MHz. In this paper, three ($n_{c}=3$) different ship target classes are considered. The maximum and minimum number of particles used in all the simulations are $L_{\max}=2000$ and $L_{\min}=300$, respectively. The CAD models and the corresponding 3D-SCMs (denoted by a red asterisk “*”) are shown in Figure 2.

### 5.1. SCM-Based JTC Method

In this simulation, the SCM-based JTC method will be evaluated under the scenario of single-target measurement without clutter. The process noise of the target motion state is characterized by $q=0.5\;m/s^{2}$. The three ship targets have the same motion model, and the initial state is $x_{0}={\left[1.2\;\mathrm{km}\;1.5\;\mathrm{km}\;-7.5\;m/s\;5.0\;m/s\right]}^{T}$. The sampling interval is t=1s and the total duration is 40 s. At a certain time, only one ship target is present, and the JTC performance of different target is analyzed separately.

**Figure 3:**
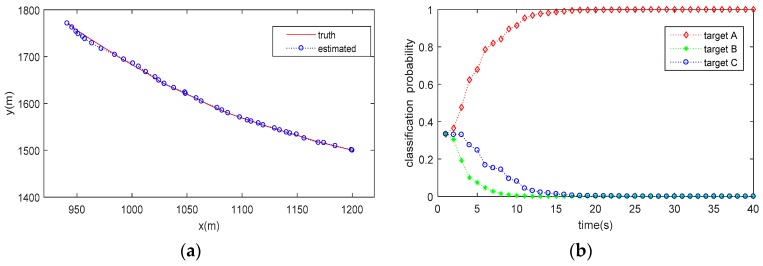
Result of JTC when Target Class A is present: (**a**) estimated target trajectory; (**b**) estimated target class probability.

**Figure 4:**
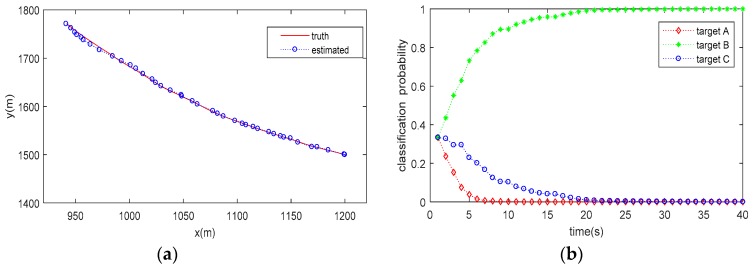
Result of JTC when Target Class B is present: (**a**) estimated target trajectory; (**b**) estimated target class probability.

**Figure 5:**
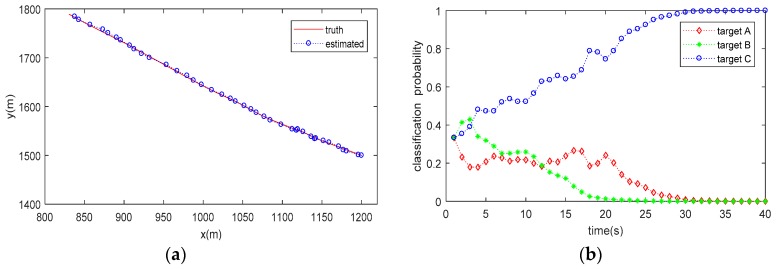
Result of JTC when Target Class C is present: (**a**) estimated target trajectory; (**b**) estimated target class probability.

The trajectory tracking (for a single run) and target classification (averaged by 20 Monte Carlo runs) results are shown in Figure 3, Figure 4 and Figure 5. As can be seen from the figures, the proposed method can not only accurately estimate the state of the target but also correctly classify the target simultaneously. It is also seen that the classification probability curve, which matches with the true target class, increases rapidly, and can approach one (100%) within 30 s under all the tested conditions. Conversely, the classification probability curves mismatching the true target class decrease gradually and reach 0 after about 10–30 estimate cycles, meaning that a high confidence classification result is obtained. However, it does not mean that we have to wait for a period of 10–30 estimate cycles to obtain a reliable decision on target class, since the classification probability matching the true target class is obviously higher than others within only a few cycles.

As a comparison, the simulation also considers the target classification result directly obtained from the HRRP correlation method, where the HRRP templates (training data) are generated from the CAD model with electromagnetic simulation tool, and the test data are predicted from the 3D-SCM. In the simulation, for each target, 360 HRRP training samples (which cover 0–360° in azimuth angle space) are generated with 1° interval. Accordingly, 1440 test samples are generated from each 3D-SCM at the same viewing angle with azimuth angle interval 0.2°. The classification results are shown in Table 1. The corresponding probabilities of correct classification (PCC) for Target Classes A, B and C are 0.8986, 0.8993 and 0.8417, respectively. The over-all PCC (OA-PCC) for all the test samples is 0.8799. The metric index PCC and OA-PCC are defined as

(93) $$PCC(m)=N_{correct}(m)/N_{total}(m),m=1,2,3.$$

(94) $$OA-PCC=\sum_{m=1}^{3}N_{correct}(m)/\sum_{m=1}^{3}N_{total}(m)$$

where $N_{total}(m)$ denotes the total test samples for the *m*th target class, $N_{correct}(m)$ represents the correctly classified test samples for the *m*th target class.

Compared the classification results in Table 1 (no tracking process involved) with those shown in Figure 3, Figure 4 and Figure 5 (with JTC processing), it is seen that the SCM-based JTC method achieves a performance improvement of more than 0.1 (10%) in PCC after the tracking filter is stable, indicating the advantage of the proposed method in classification accuracy.

### 5.2. SCM-JTC-CBMeMBer Filter

In this simulation, all three classes of ship targets will appear in the surveillance area. Target A appears at time k=5 and disappears at time k=55 with initial state $x_{0}^{\left(1\right)}={\left[1000m\;1000m\;-9.82m/s\;-9.82m/s\right]}^{T}$. Target B appears at time k=15 and disappears at time k=65 with initial state $x_{0}^{\left(2\right)}={\left[1000m\;-1000m\;-9.82m/s\;9.82m/s\right]}^{T}$. Target C appears at time k=25 and disappears at time k=75 with initial state $x_{0}^{\left(3\right)}={\left[-1000m\;-1000m\;9.82m/s\;9.82m/s\right]}^{T}$. The Poisson average clutter rate is $\lambda_{c}=3$. The surveillance area is [−2000m,2000m]×[−2000m,2000m]. Target surviving probability and detection probability are $p_{S,k}=p_{D,k}=0.99$. The sampling interval t is 1 s and the total simulation time is 100 s. The target births are modeled as multi-Bernoulli RFS with $\pi_{B}={\left\{\left(r_{B}^{\left(i\right)},p_{B}^{\left(i\right)}(x|Z)\right)\right\}}_{i=1}^{4}$, where

(95) $$\begin{array}{l} r_{B}^{\left(1\right)}=r_{B}^{\left(2\right)}=r_{B}^{\left(3\right)}=r_{B}^{\left(4\right)}=0.02\\ p_{B}^{\left(i\right)}(c^{1}|Z)=p_{B}^{\left(i\right)}(c^{2}|Z)=p_{B}^{\left(i\right)}(c^{3}|Z)=1/3\\ p_{B}^{\left(i\right)}(x|Z)=N(x;m^{\left(i\right)},P^{\left(i\right)})\\ P^{\left(1\right)}=P^{\left(2\right)}=P^{\left(3\right)}=P^{\left(4\right)}=diag([100m^{2}\;100m^{2}\;10m^{2}{/s}^{2}\;10m^{2}/s^{2}])\\ m^{\left(1\right)}={\left[1000m\;1000m\;-10m/s\;-10m/s\right]}^{T}\\ m^{\left(2\right)}={\left[1000m\;-1000m\;-10m/s\;10m/s\right]}^{T}\\ m^{\left(3\right)}={\left[-1000m\;-1000m\;10m/s\;10m/s\right]}^{T}\\ m^{\left(4\right)}={\left[-1000m\;1000m\;10m/s\;-10m/s\right]}^{T} \end{array}$$

**Figure 6:**
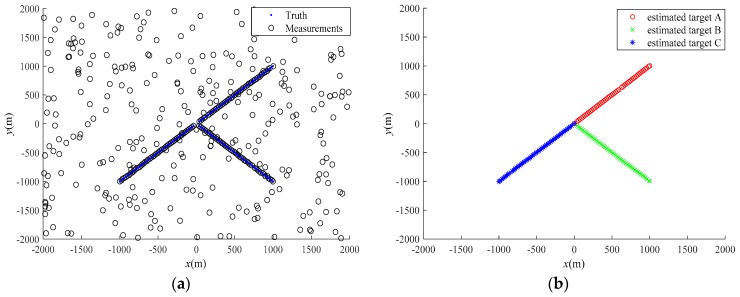
Multi-target tracking results for a single run: (**a**) the true target trajectories and received measurements; (**b**) estimated target trajectories.

The trajectory tracking results for a single run are shown in Figure 6. As can be clearly seen from Figure 6, under the clutter environments, the proposed SCM-JTC-CBMeMBer filter can estimate target number and state correctly, and it can also obtain correct target classification, which will be further validated by the repeated Monte Carlo trials.

To further test the performance of the proposed SCM-JTC-CBMeBer filter, 50 Monte Carlo runs are carried out under the same scenario as above. Specifically, the metric of Optimal Subpattern Assignment (OSPA) distance [34] is used to evaluate the multi-target tracking results, and the CBMeMBer filter is also considered as a comparison.

**Figure 7:**
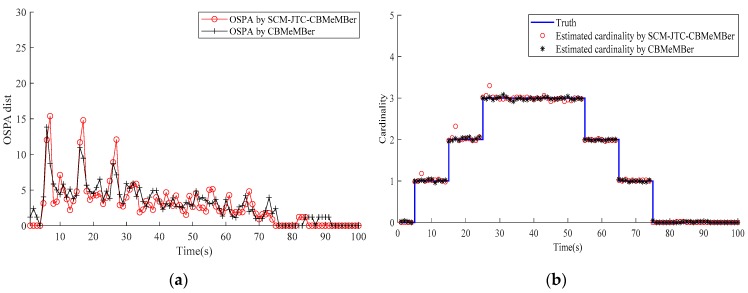
Estimated target state: (**a**) Optimal Subpattern Assignment (OSPA) distance; (**b**) the estimated cardinality.

**Figure 8:**
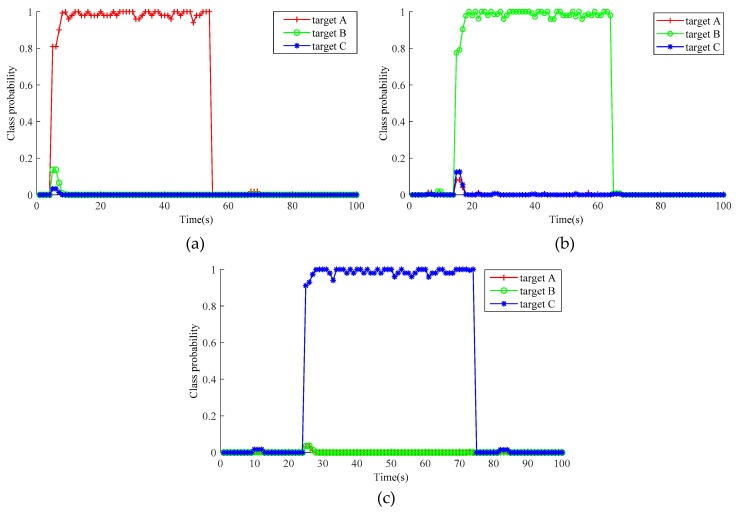
Results of targets classification: (**a**) Ship Target A; (**b**) Ship Target B; (**c**) Ship Target C.

The OSPA distance and cardinality estimation are shown in Figure 7, and the target classification results are plotted in Figure 8. 

From Figure 7, we can see that the SCM-JTC-CBMeMBer filter can effectively estimate the target state and target number. At the instant when the target appears and disappears, a slight degradation in estimation performance is observed, which is the normal phenomenon confronted in multi-target tracking. Compared with the conventional CBMeMBer filter (which can only be used for multi-target tracking purposes rather than targets classification), the SCM-JTC-CBMeMBer filter has almost the same performance in target tracking. 

As can be seen from Figure 8, the SCM-JTC-CBMeMBer filter can also correctly classify multiple targets, and the classification probability of each target is very high (almost reaches one). 

## 6. Conclusions

In this paper, an SCM-based JTC method is first introduced. The presented method can implement target tracking and classification simultaneously by using a model based on HRRP prediction, target kinematic and HRRP measurements, and thus to alleviate the dependence of target classification on the requirements of target maneuvers or other support information (such as target attribute/identity) in the conventional methods. The SCM-based JTC is then integrated into the framework of the CBMeMBer filter, and the resulting SCM-JTC-CBMeMBer filter for multi-target JTC is derived under the condition with detection uncertainty. Finally, the SMC technique is adopted to implement the proposed filter in view of the complex calculation in multi-target state recursion. Simulations are carried out under the typical scenario with three different ship targets, and the results show that the developed method can not only effectively estimate the target state, but also obtain reliable target classification decision. Additionally, the proposed joint processing method can achieve better performance than separate HRRP classification without involving target tracking. 

## Figures and Tables

**Table 1:** Classification results of the ship targets.

True Target Class	Classified Results			PCC
	**Ship A**	Ship B	Ship C	
Ship A	**1294**	58	99	**0.8986**
Ship B	77	**1295**	129	**0.8993**
Ship C	69	87	**1212**	**0.8417**
OA-PCC	**0.8799**			

## Appendix A

According to Equations (14), (15) and (42), we can obtain

(A1) $$\begin{array}{ll} r_{P,k|k-1}^{\left(i\right)} & =r_{k-1}^{\left(i\right)}\langle p_{k-1}^{\left(i\right)}(x_{k-1}),p_{S,k}\rangle \\ & =r_{k-1}^{\left(i\right)}\langle \sum_{m=1}^{n_{c}}p_{k-1}^{\left(i\right)}(x_{k-1}|c^{m},Z^{k-1})p_{k-1}^{\left(i\right)}(c^{m}|Z^{k-1}),p_{S,k}\rangle \\ & =r_{k-1}^{\left(i\right)}\sum_{m=1}^{n_{c}}p_{k-1}^{\left(i\right)}(c^{m}|Z^{k-1})\langle p_{k-1}^{\left(i\right)}(x_{k-1}|c^{m},Z^{k-1}),p_{S,k}\rangle \end{array}$$

(A2) $$\begin{array}{ll} p_{P,k|k-1}^{\left(i\right)}(x_{k}|Z^{k-1}) & =\frac{\langle f_{k|k-1}(x_{k}|x_{k-1}),p_{k-1}^{\left(i\right)}(x_{k-1})p_{S,k}\rangle }{\langle p_{k-1}^{\left(i\right)}(x_{k-1}),p_{S,k}\rangle }\\ & =\frac{\langle f_{k|k-1}^{k}(x_{k}|x_{k-1}),\sum_{m=1}^{n_{c}}p_{k-1}^{\left(i\right)}(x_{k-1}|c^{m},Z^{k-1})p_{k-1}^{\left(i\right)}(c^{m}|Z^{k-1})p_{S,k}\rangle }{\langle \sum_{m=1}^{n_{c}}p_{k-1}^{\left(i\right)}(x_{k-1}|c^{m},Z^{k-1})p_{k-1}^{\left(i\right)}(c^{m}|Z^{k-1}),p_{S,k}\rangle }\\ & =\frac{\sum_{m=1}^{n_{c}}p_{k-1}^{\left(i\right)}(c^{m}|Z^{k-1})\langle f_{k|k-1}^{k}(x_{k}|x_{k-1}),p_{k-1}^{\left(i\right)}(x_{k-1}|c^{m},Z^{k-1})p_{S,k}\rangle }{\sum_{m=1}^{n_{c}}p_{k-1}^{\left(i\right)}(c^{m}|Z^{k-1})\langle p_{k-1}^{\left(i\right)}(x_{k-1}|c^{m},Z^{k-1}),p_{S,k}\rangle } \end{array}$$

## Appendix B

According to Equations (17)–(20) and Equation (50), we have

(A3) $$\begin{array}{ll} r_{L,k}^{\left(i\right)} & =r_{k|k-1}^{\left(i\right)}\frac{1-\langle p_{k|k-1}^{\left(i\right)}(x_{k}),p_{D,k}\rangle }{1-r_{k|k-1}^{\left(i\right)}\langle p_{k|k-1}^{\left(i\right)}(x_{k}),p_{D,k}\rangle }\\ & =r_{k|k-1}^{\left(i\right)}\frac{1-\langle \sum_{m=1}^{n_{c}}p_{k|k-1}^{\left(i\right)}(x_{k}|c^{m},Z^{k-1})p_{k|k-1}^{\left(i\right)}(c^{m}|Z^{k-1}),p_{D,k}\rangle }{1-r_{k|k-1}^{\left(i\right)}\langle \sum_{m=1}^{n_{c}}p_{k|k-1}^{\left(i\right)}(x_{k}|c^{m},Z^{k-1})p_{k|k-1}^{\left(i\right)}(c^{m}|Z^{k-1}),p_{D,k}\rangle }\\ & =r_{k|k-1}^{\left(i\right)}\frac{1-\sum_{m=1}^{n_{c}}p_{k|k-1}^{\left(i\right)}(c^{m}|Z^{k-1})\langle p_{k|k-1}^{\left(i\right)}(x_{k}|c^{m},Z^{k-1}),p_{D,k}\rangle }{1-r_{k|k-1}^{\left(i\right)}\sum_{m=1}^{n_{c}}p_{k|k-1}^{\left(i\right)}(c^{m}|Z^{k-1})\langle p_{k|k-1}^{\left(i\right)}(x_{k}|c^{m},Z^{k-1}),p_{D,k}\rangle } \end{array}$$

(A4) $$\begin{array}{ll} p_{L,k}^{\left(i\right)}(x_{k} & |Z^{k})=p_{k|k-1}^{\left(i\right)}(x_{k}|Z^{k-1})\frac{1-p_{D,k}}{1-\langle p_{k|k-1}^{\left(i\right)}(x_{k}|Z^{k-1}),p_{D,k}\rangle }\\ & =\sum_{m=1}^{n_{c}}p_{k|k-1}^{\left(i\right)}(x_{k}|c^{m},Z^{k-1})p_{k|k-1}^{\left(i\right)}(c^{m}|Z^{k-1})\frac{1-p_{D,k}}{1-\langle \sum_{m=1}^{n_{c}}p_{k|k-1}^{\left(i\right)}(x_{k}|c^{m},Z^{k-1})p_{k|k-1}^{\left(i\right)}(c^{m}|Z^{k-1}),p_{D,k}\rangle }\\ & =\sum_{m=1}^{n_{c}}p_{k|k-1}^{\left(i\right)}(c^{m}|Z^{k-1})\frac{1-p_{D,k}}{1-\sum_{m=1}^{n_{c}}p_{k|k-1}^{\left(i\right)}(c^{m}|Z^{k-1})\langle p_{k|k-1}^{\left(i\right)}(x_{k}|c^{m},Z^{k-1}),p_{D,k}\rangle }p_{k|k-1}^{\left(i\right)}(x_{k}|c^{m},Z^{k-1}) \end{array}$$

(A5) $$\begin{array}{ll} r_{U,k}^{*}(\tilde{z}) & =\frac{\sum_{i=1}^{M_{k|k-1}}\frac{r_{k|k-1}^{\left(i\right)}(1-r_{k|k-1}^{\left(i\right)})\langle p_{k|k-1}^{\left(i\right)}(x_{k}|Z^{k-1}),g_{k}(\tilde{z}|x_{k})p_{D,k}\rangle }{{\left(1-r_{k|k-1}^{\left(i\right)}\langle p_{k|k-1}^{\left(i\right)}(x_{k}|Z^{k-1}),p_{D,k}\rangle \right)}^{2}}}{\kappa (\tilde{z})+\sum_{i=1}^{M_{k|k-1}}\frac{r_{k|k-1}^{\left(i\right)}\langle p_{k|k-1}^{\left(i\right)}(x_{k}|Z^{k-1}),g_{k}(\tilde{z}|x_{k})p_{D,k}\rangle }{1-r_{k|k-1}^{\left(i\right)}\langle p_{k|k-1}^{\left(i\right)}(x_{k}|Z^{k-1}),p_{D,k}\rangle }}\\ & =\frac{\sum_{i=1}^{M_{k|k-1}}\frac{r_{k|k-1}^{\left(i\right)}(1-r_{k|k-1}^{\left(i\right)})\langle \sum_{m=1}^{n_{c}}p_{k|k-1}^{\left(i\right)}(x_{k}|c^{m},Z^{k-1})p_{k|k-1}^{\left(i\right)}(c^{m}|Z^{k-1}),g_{k}^{k}(x_{k})g_{k}^{c}(x_{k},c_{}^{m})p_{D,k}\rangle }{{\left(1-r_{k|k-1}^{\left(i\right)}\langle \sum_{m=1}^{n_{c}}p_{k|k-1}^{\left(i\right)}(x_{k}|c^{m},Z^{k-1})p_{k|k-1}^{\left(i\right)}(c^{m}|Z^{k-1}),p_{D,k}\rangle \right)}^{2}}}{\kappa (\tilde{z})+\sum_{i=1}^{M_{k|k-1}}\frac{r_{k|k-1}^{\left(i\right)}\langle \sum_{m=1}^{n_{c}}p_{k|k-1}^{\left(i\right)}(x_{k}|c^{m},Z^{k-1})p_{k|k-1}^{\left(i\right)}(c^{m}|Z^{k-1}),g_{k}^{k}(x_{k})g_{k}^{c}(x_{k},c_{}^{m})p_{D,k}\rangle }{1-r_{k|k-1}^{\left(i\right)}\langle \sum_{m=1}^{n_{c}}p_{k|k-1}^{\left(i\right)}(x_{k}|c^{m},Z^{k-1})p_{k|k-1}^{\left(i\right)}(c^{m}|Z^{k-1}),p_{D,k}\rangle }}\\ & =\frac{\sum_{i=1}^{M_{k|k-1}}\frac{r_{k|k-1}^{\left(i\right)}(1-r_{k|k-1}^{\left(i\right)})\sum_{m=1}^{n_{c}}p_{k|k-1}^{\left(i\right)}(c^{m}|Z^{k-1})\langle p_{k|k-1}^{k,(i)}(x_{k}|c^{m},Z^{k-1}),g_{k}^{k}(x_{k})g_{k}^{c}(x_{k},c_{}^{m})p_{D,k}\rangle }{{\left(1-r_{k|k-1}^{\left(i\right)}\sum_{m=1}^{n_{c}}p_{k|k-1}^{\left(i\right)}(c^{m}|Z^{k-1})\langle p_{k|k-1}^{\left(i\right)}(x_{k}|c^{m},Z^{k-1}),p_{D,k}\rangle \right)}^{2}}}{\kappa (\tilde{z})+\sum_{i=1}^{M_{k|k-1}}\frac{r_{k|k-1}^{\left(i\right)}\sum_{m=1}^{n_{c}}p_{k|k-1}^{\left(i\right)}(c^{m}|Z^{k-1})\langle p_{k|k-1}^{\left(i\right)}(x_{k}|c^{m},Z^{k-1}),g_{k}^{k}(x_{k})g_{k}^{c}(x_{k},c_{}^{m})p_{D,k}\rangle }{1-r_{k|k-1}^{\left(i\right)}\sum_{m=1}^{n_{c}}p_{k|k-1}^{\left(i\right)}(c^{m}|Z^{k-1})\langle p_{k|k-1}^{k,(i)}(x_{k}|c^{m},Z^{k-1}),p_{D,k}\rangle }} \end{array}$$

(A6) $$\begin{array}{ll} p_{U,k}^{*}(x_{k};\tilde{z}) & =\frac{\sum_{i=1}^{M_{k|k-1}}\frac{r_{k|k-1}^{\left(i\right)}p_{k|k-1}^{\left(i\right)}(x_{k}|Z^{k-1})p_{D,k}g_{k}^{k}(x_{k})g_{k}^{c}(x_{k},c_{}^{m})}{1-r_{k|k-1}^{\left(i\right)}}}{\sum_{i=1}^{M_{k|k-1}}\frac{r_{k|k-1}^{\left(i\right)}}{1-r_{k|k-1}^{\left(i\right)}}\langle p_{k|k-1}^{\left(i\right)}(x_{k}|Z^{k-1}),p_{D,k}g_{k}^{k}(x_{k})g_{k}^{c}(x_{k},c^{m})\rangle }\\ & =\frac{\sum_{i=1}^{M_{k|k-1}}\frac{r_{k|k-1}^{\left(i\right)}\sum_{m=1}^{n_{c}}p_{k|k-1}^{\left(i\right)}(x_{k}|c^{m},Z^{k-1})p_{k|k-1}^{\left(i\right)}(c^{m}|Z^{k-1})p_{D,k}g_{k}^{k}(x_{k})g_{k}^{c}(x_{k},c_{}^{m})}{1-r_{k|k-1}^{\left(i\right)}}}{\sum_{i=1}^{M_{k|k-1}}\frac{r_{k|k-1}^{\left(i\right)}}{1-r_{k|k-1}^{\left(i\right)}}\langle \sum_{m=1}^{n_{c}}p_{k|k-1}^{\left(i\right)}(x_{k}|c^{m},Z^{k-1})p_{k|k-1}^{\left(i\right)}(c^{m}|Z^{k-1}),p_{D,k}g_{k}^{k}(x_{k})g_{k}^{c}(x_{k},c_{}^{m})\rangle }\\ & =\frac{\sum_{m=1}^{n_{c}}\sum_{i=1}^{M_{k|k-1}}\frac{r_{k|k-1}^{\left(i\right)}p_{k|k-1}^{\left(i\right)}(x_{k}|c^{m},Z^{k-1})p_{k|k-1}^{\left(i\right)}(c^{m}|Z^{k-1})p_{D,k}g_{k}^{k}(x_{k})g_{k}^{c}(x_{k},c_{}^{m})}{1-r_{k|k-1}^{\left(i\right)}}}{\sum_{i=1}^{M_{k|k-1}}\frac{r_{k|k-1}^{\left(i\right)}}{1-r_{k|k-1}^{\left(i\right)}}\langle \sum_{m=1}^{n_{c}}p_{k|k-1}^{\left(i\right)}(x_{k}|c^{m},Z^{k-1})p_{k|k-1}^{\left(i\right)}(c^{m}|Z^{k-1}),p_{D,k}g_{k}^{k}(x_{k})g_{k}^{c}(x_{k},c_{}^{m})\rangle } \end{array}$$

## Appendix C

According to Equations (44)–(49) and Equation (58), we can obtain

(A7) $$\begin{array}{ll} r_{P,k|k-1}^{\left(i\right)} & =r_{k-1}^{\left(i\right)}\sum_{m=1}^{n_{c}}p_{k-1}^{\left(i\right)}(c^{m}|Z^{k-1})\langle p_{k-1}^{\left(i\right)}(x_{k-1}|c^{m},Z^{k-1}),p_{S,k}\rangle \\ & =r_{k-1}^{\left(i\right)}\sum_{m=1}^{n_{c}}\frac{\sum_{j=1}^{n_{k-1}^{i}}w_{k-1}^{i,j}\delta_{l^{i,j}}(c^{m})}{\sum_{j=1}^{n_{k-1}^{i}}w_{k-1}^{i,j}}\langle \sum_{j=1}^{n_{k-1}^{i}}w_{k-1}^{i,m,j}\delta_{x_{k-1}^{i,m,j}}(x_{k-1}),p_{S,k}\rangle \\ & =r_{k-1}^{\left(i\right)}\sum_{m=1}^{n_{c}}\frac{\sum_{j=1}^{n_{k-1}^{i}}w_{k-1}^{i,j}\delta_{c^{i,j}}(m)}{\sum_{j=1}^{n_{k-1}^{i}}w_{k-1}^{i,j}}\langle \sum_{j=1}^{n_{k-1}^{i}}\frac{w_{k-1}^{i,j}\delta_{c^{i,j}}(m)}{\sum_{j=1}^{n_{k-1}^{i}}w_{k-1}^{i,j}\delta_{c^{i,j}}(m)}\delta_{x_{k-1}^{i,m,j}}(x),p_{S,k}\rangle \\ & =r_{k-1}^{\left(i\right)}\sum_{m=1}^{n_{c}}\sum_{j=1}^{n_{k-1}^{i}}\frac{\sum_{j=1}^{n_{k-1}^{i}}w_{k-1}^{i,j}\delta_{l^{i,j}}(c^{m})}{\sum_{j=1}^{n_{k-1}^{i}}w_{k-1}^{i,j}\delta_{l^{i,j}}(c^{m})}w_{k-1}^{i,j}\delta_{l^{i,j}}(c^{m})p_{S,k}(x_{k-1}^{i,m,j})\\ & =r_{k-1}^{\left(i\right)}\sum_{j=1}^{n_{k-1}^{i}}w_{k-1}^{i,j}p_{S,k} \end{array}$$

(A8) $$p_{P,k|k-1}^{\left(i\right)}(c^{m}|Z)=p_{k-1}^{\left(i\right)}(c^{m}|Z^{k-1})=\frac{\sum_{j=1}^{n_{k-1}^{i}}w_{P,k|k-1}^{i,j}\delta_{l^{i,j}}(c^{m})}{\sum_{j=1}^{n_{k-1}^{i}}w_{P,k|k-1}^{i,j}}$$

(A9) $$\begin{array}{ll} p_{P,k|k-1}^{\left(i\right)}(x_{k} & \left|c^{m},Z^{k-1}\right)\\ & =\frac{\langle f_{k|k-1}^{k}(x_{k}|x_{k-1}),p_{k-1}^{\left(i\right)}(x_{k-1}|c^{m},Z^{k-1})p_{S,k}\rangle }{\sum_{m=1}^{n_{c}}p_{k-1}^{\left(i\right)}(c^{m}|Z^{k-1})\langle p_{k-1}^{\left(i\right)}(x_{k-1}|c^{m},Z^{k-1}),p_{S,k}\rangle }\\ & =\frac{\langle f_{k|k-1}^{k}(x_{k}|x_{k-1}),\sum_{j=1}^{n_{k-1}^{i}}w_{k-1}^{i,m,j}\delta_{x_{k-1}^{i,m,j}}(x_{k-1})p_{S,k}\rangle }{\sum_{m=1}^{n_{c}}\frac{\sum_{j=1}^{n_{k-1}^{i}}w_{k-1}^{i,j}\delta_{l^{i,j}}(c^{m})}{\sum_{j=1}^{n_{k-1}^{i}}w_{k-1}^{i,j}}\langle \sum_{j=1}^{n_{k-1}^{i}}w_{k-1}^{i,m,j}\delta_{x_{k-1}^{i,m,j}}(x),p_{S,k}\rangle }\\ & =\frac{\sum_{j=1}^{n_{k-1}^{i}}w_{k-1}^{i,m,j}p_{S,k}(x_{k-1}^{i,m,j})\delta_{x_{P,k|k-1}^{i,m,j}}(x_{k})}{\sum_{m=1}^{n_{c}}\frac{\sum_{j=1}^{n_{k-1}^{i}}w_{k-1}^{i,j}\delta_{l^{i,j}}(c^{m})}{\sum_{j=1}^{n_{k-1}^{i}}w_{k-1}^{i,j}}\sum_{j=1}^{n_{k-1}^{i}}w_{k-1}^{i,m,j}p_{S,k}(x_{k-1}^{i,m,j})}=\frac{\sum_{j=1}^{n_{k-1}^{i}}w_{k-1}^{i,m,j}p_{S,k}\delta_{x_{P,k|k-1}^{i,m,j}}(x)}{\sum_{j=1}^{n_{k-1}^{i}}w_{k-1}^{i,j}p_{S,k}} \end{array}$$

## Appendix D

According to Equations (52)–(57), we have

(A10) $$\begin{array}{ll} r_{L,k}^{\left(i\right)} & =r_{k|k-1}^{\left(i\right)}\frac{1-\sum_{m=1}^{n_{c}}p_{k|k-1}^{\left(i\right)}(c^{m}|Z^{k-1})\langle p_{k|k-1}^{\left(i\right)}(x_{k}|c^{m},Z^{k-1}),p_{D,k}\rangle }{1-r_{k|k-1}^{\left(i\right)}\sum_{m=1}^{n_{c}}p_{k|k-1}^{\left(i\right)}(c^{m}|Z^{k-1})\langle p_{k|k-1}^{\left(i\right)}(x_{k}|c^{m},Z^{k-1}),p_{D,k}\rangle }\\ & =r_{k|k-1}^{\left(i\right)}\frac{1-\sum_{m=1}^{n_{c}}\frac{\sum_{j=1}^{n_{k-1}^{i}}w_{k|k-1}^{i,j}\delta_{l^{i,j}}(c^{m})}{\sum_{j=1}^{n_{k-1}^{i}}w_{k|k-1}^{i,j}}\langle \sum_{j=1}^{n_{k-1}^{i}}w_{k|k-1}^{i,m,j}\delta_{x_{k|k-1}^{i,m,j}}(x_{k}),p_{D,k}\rangle }{1-r_{k|k-1}^{\left(i\right)}\sum_{m=1}^{n_{c}}\frac{\sum_{j=1}^{n_{k-1}^{i}}w_{k|k-1}^{i,j}\delta_{l^{i,j}}(c^{m})}{\sum_{j=1}^{n_{k-1}^{i}}w_{k|k-1}^{i,j}}\langle \sum_{j=1}^{n_{k-1}^{i}}w_{k|k-1}^{i,m,j}\delta_{x_{k|k-1}^{i,m,j}}(x_{k}),p_{D,k}\rangle }\\ & =r_{k|k-1}^{\left(i\right)}\frac{1-\sum_{j=1}^{n_{k-1}^{i}}w_{k|k-1}^{i,j}p_{D,k}}{1-r_{k|k-1}^{\left(i\right)}\sum_{j=1}^{n_{k-1}^{i}}w_{k|k-1}^{i,j}p_{D,k}} \end{array}$$

(A11) $$\begin{array}{ll} p_{L,k}^{\left(i\right)}(x_{k}|c^{m},Z^{k}) & =\frac{1-p_{D,k}}{1-\sum_{m=1}^{n_{c}}p_{k|k-1}^{\left(i\right)}(c^{m}|Z^{k-1})\langle p_{k|k-1}^{\left(i\right)}(x_{k}|c^{m},Z^{k-1}),p_{D,k}\rangle }p_{k|k-1}^{\left(i\right)}(x_{k}|c^{m},Z^{k-1})\\ & =\frac{1-p_{D,k}}{1-\sum_{m=1}^{n_{c}}\frac{\sum_{j=1}^{n_{k-1}^{i}}w_{k|k-1}^{i,j}\delta_{l^{i,j}}(c^{m})}{\sum_{j=1}^{n_{k-1}^{i}}w_{k|k-1}^{i,j}}\langle \sum_{j=1}^{n_{k-1}^{i}}w_{k|k-1}^{i,m,j}\delta_{x_{k|k-1}^{i,m,j}}(x_{k}),p_{D,k}\rangle }\sum_{j=1}^{n_{k-1}^{i}}w_{k|k-1}^{i,m,j}\delta_{x_{k|k-1}^{i,m,j}}(x_{k})\\ & =\frac{1-p_{D,k}}{1-\sum_{m=1}^{n_{c}}\sum_{j=1}^{n_{k-1}^{i}}\frac{\sum_{j=1}^{n_{k-1}^{i}}w_{k|k-1}^{i,j}\delta_{l^{i,j}}(c^{m})}{\sum_{j=1}^{n_{k-1}^{i}}w_{k|k-1}^{i,j}}\frac{w_{k|k-1}^{i,j}\delta_{l^{i,j}}(c^{m})}{\sum_{j=1}^{n_{k-1}^{i}}w_{k|k-1}^{i,j}\delta_{l^{i,j}}(c^{m})}p_{D,k}(x_{k|k-1}^{i,m,j})}\sum_{j=1}^{n_{k-1}^{i}}w_{k|k-1}^{i,m,j}\delta_{x_{k|k-1}^{i,m,j}}(x_{k})\\ & =\frac{1-p_{D,k}}{1-\sum_{j=1}^{n_{k-1}^{i}}w_{k|k-1}^{i,j}p_{D,k}}\sum_{j=1}^{n_{k-1}^{i}}w_{k|k-1}^{i,m,j}\delta_{x_{k|k-1}^{i,m,j}}(x_{k}) \end{array}$$

(A12) $$\begin{array}{ll} r_{U,k}^{*}(\tilde{z}) & =\frac{\sum_{i=1}^{M_{k|k-1}}\frac{r_{k|k-1}^{\left(i\right)}(1-r_{k|k-1}^{\left(i\right)})\sum_{m=1}^{n_{c}}p_{k|k-1}^{\left(i\right)}(c^{m}|Z^{k-1})\langle p_{k|k-1}^{\left(i\right)}(x_{k}|c^{m},Z^{k-1}),g_{k}^{k}(x_{k})g_{k}^{c}(x_{k},c^{m})p_{D,k}\rangle }{{\left(1-r_{k|k-1}^{\left(i\right)}\sum_{m=1}^{n_{c}}p_{k|k-1}^{\left(i\right)}(c^{m}|Z^{k-1})\langle p_{k|k-1}^{\left(i\right)}(x_{k}|c^{m},Z^{k-1}),p_{D,k}\rangle \right)}^{2}}}{\kappa_{k}(\tilde{z})+\sum_{i=1}^{M_{k|k-1}}\frac{r_{k|k-1}^{\left(i\right)}\sum_{m=1}^{n_{c}}p_{k|k-1}^{\left(i\right)}(c^{m}|Z^{k-1})\langle p_{k|k-1}^{\left(i\right)}(x_{k}|c^{m},Z^{k-1}),g_{k}^{k}(x_{k})g_{k}^{c}(x_{k},c^{m})p_{D,k}\rangle }{1-r_{k|k-1}^{\left(i\right)}\sum_{m=1}^{n_{c}}p_{k|k-1}^{\left(i\right)}(c^{m}|Z^{k-1})\langle p_{k|k-1}^{\left(i\right)}(x_{k}|c^{m},Z^{k-1}),p_{D,k}\rangle }}\\ & =\frac{\sum_{i=1}^{M_{k|k-1}}\frac{r_{k|k-1}^{\left(i\right)}(1-r_{k|k-1}^{\left(i\right)})\sum_{m=1}^{n_{c}}\frac{\sum_{j=1}^{n_{k-1}^{i}}w_{k|k-1}^{i,j}\delta_{l^{i,j}}(c^{m})}{\sum_{j=1}^{n_{k-1}^{i}}w_{k|k-1}^{i,j}}\langle \sum_{j=1}^{n_{k-1}^{i}}w_{k|k-1}^{i,m,j}\delta_{x_{k|k-1}^{i,m,j}}(x_{k}),g_{k}^{k}(x_{k})g_{k}^{c}(x_{k},c^{m})p_{D,k}\rangle }{{\left(1-r_{k|k-1}^{\left(i\right)}\sum_{m=1}^{n_{c}}\frac{\sum_{j=1}^{n_{k-1}^{i}}w_{k|k-1}^{i,j}\delta_{l^{i,j}}(c^{m})}{\sum_{j=1}^{n_{k-1}^{i}}w_{k|k-1}^{i,j}}\langle \sum_{j=1}^{n_{k-1}^{i}}w_{k|k-1}^{i,m,j}\delta_{x_{k|k-1}^{i,m,j}}(x),p_{D,k}\rangle \right)}^{2}}}{\kappa (\tilde{z})+\sum_{i=1}^{M_{k|k-1}}\frac{r_{k|k-1}^{\left(i\right)}\sum_{m=1}^{n_{c}}\frac{\sum_{j=1}^{n_{k-1}^{i}}w_{k|k-1}^{i,j}\delta_{l^{i,j}}(c^{m})}{\sum_{j=1}^{n_{k-1}^{i}}w_{k|k-1}^{i,j}}\langle \sum_{j=1}^{n_{k-1}^{i}}w_{k|k-1}^{i,m,j}\delta_{x_{k|k-1}^{i,m,j}}(x_{k}),g_{k}^{k}(x_{k})g_{k}^{c}(x_{k},c^{m})p_{D,k}\rangle }{1-r_{k|k-1}^{\left(i\right)}\sum_{m=1}^{n_{c}}\frac{\sum_{j=1}^{n_{k-1}^{i}}w_{k|k-1}^{i,j}\delta_{l^{i,j}}(c^{m})}{\sum_{j=1}^{n_{k-1}^{i}}w_{k|k-1}^{i,j}}\langle \sum_{j=1}^{n_{k-1}^{i}}w_{k|k-1}^{i,m,j}\delta_{x_{k|k-1}^{i,m,j}}(x_{k}),p_{D,k}\rangle }}\\ & =\frac{\sum_{i=1}^{M_{k|k-1}}\frac{r_{k|k-1}^{\left(i\right)}(1-r_{k|k-1}^{\left(i\right)})\sum_{j=1}^{n_{k-1}^{i}}w_{k|k-1}^{i,j}g_{k}^{k}(x_{k|k-1}^{i,j})g_{k}^{c}(x_{k|k-1}^{i,j},c^{m})p_{D,k}}{{\left(1-r_{k|k-1}^{\left(i\right)}\sum_{j=1}^{n_{k-1}^{i}}w_{k|k-1}^{i,j}p_{D,k}\right)}^{2}}}{\kappa (\tilde{z})+\sum_{i=1}^{M_{k|k-1}}\frac{r_{k|k-1}^{\left(i\right)}\sum_{j=1}^{n_{k-1}^{i}}w_{k|k-1}^{i,j}g_{k}^{k}(x_{k|k-1}^{i,j})g_{k}^{c}(x_{k|k-1}^{i,j},c^{m})p_{D,k}}{1-r_{k|k-1}^{\left(i\right)}\sum_{j=1}^{n_{k-1}^{i}}w_{k|k-1}^{i,j}p_{D,k}}}\\ & =\frac{\sum_{i=1}^{M_{k|k-1}}\frac{r_{k|k-1}^{\left(i\right)}(1-r_{k|k-1}^{\left(i\right)})\sum_{j=1}^{n_{k-1}^{i}}w_{k|k-1}^{i,j}g_{k}^{k}(x_{k|k-1}^{i,j})g_{k}^{c}(x_{k|k-1}^{i,j})p_{D,k}}{{\left(1-r_{k|k-1}^{\left(i\right)}\sum_{j=1}^{n_{k-1}^{i}}w_{k|k-1}^{i,j}p_{D,k}\right)}^{2}}}{\kappa (\tilde{z})+\sum_{i=1}^{M_{k|k-1}}\frac{r_{k|k-1}^{\left(i\right)}\sum_{j=1}^{n_{k-1}^{i}}w_{k|k-1}^{i,j}g_{k}^{k}(x_{k|k-1}^{i,j})g_{k}^{c}(x_{k|k-1}^{i,j})p_{D,k}}{1-r_{k|k-1}^{\left(i\right)}\sum_{j=1}^{n_{k-1}^{i}}w_{k|k-1}^{i,j}p_{D,k}}} \end{array}$$

(A13) $$\begin{array}{ll} p_{U,k}^{\left(i\right)}(x_{k}|c^{m},Z^{k}) & =\frac{\sum_{i=1}^{M_{k|k-1}}\frac{r_{k|k-1}^{\left(i\right)}p_{k|k-1}^{\left(i\right)}(x_{k}|c^{m},Z^{k-1})p_{k|k-1}^{\left(i\right)}(c^{m}|Z^{k-1})p_{D,k}g_{k}^{k}(x_{k})g_{k}^{c}(x_{k},c^{m})}{1-r_{k|k-1}^{\left(i\right)}}}{\sum_{i=1}^{M_{k|k-1}}\frac{r_{k|k-1}^{\left(i\right)}p_{k|k-1}^{\left(i\right)}(c^{m}|Z^{k-1})\langle p_{k|k-1}^{\left(i\right)}(x_{k}|c^{m},Z^{k-1}),p_{D,k}g_{k}^{k}(x_{k})g_{k}^{c}(x_{k},c^{m})\rangle }{1-r_{k|k-1}^{\left(i\right)}}}\\ & =\frac{\sum_{i=1}^{M_{k|k-1}}\frac{r_{k|k-1}^{\left(i\right)}\sum_{j=1}^{n_{k-1}^{i}}w_{k|k-1}^{i,m,j}\delta_{x_{k|k-1}^{i,m,j}}(x_{k})\frac{\sum_{j=1}^{n_{k-1}^{i}}w_{k|k-1}^{i,j}\delta_{l^{i,j}}(c^{m})}{\sum_{j=1}^{n_{k-1}^{i}}w_{k|k-1}^{i,j}}p_{D,k}g_{k}^{k}(x_{k})g_{k}^{c}(x_{k},c^{m})}{1-r_{k|k-1}^{\left(i\right)}}}{\sum_{i=1}^{M_{k|k-1}}\frac{r_{k|k-1}^{\left(i\right)}\frac{\sum_{j=1}^{n_{k-1}^{i}}w_{k|k-1}^{i,j}\delta_{l^{i,j}}(c^{m})}{\sum_{j=1}^{n_{k-1}^{i}}w_{k|k-1}^{i,j}}\langle \sum_{j=1}^{n_{k-1}^{i}}w_{k|k-1}^{i,m,j}\delta_{x_{k|k-1}^{i,m,j}}(x_{k}),p_{D,k}g_{k}^{k}(x_{k})g_{k}^{c}(x_{k},c^{m})\rangle }{1-r_{k|k-1}^{\left(i\right)}}}\\ & =\frac{\sum_{i=1}^{M_{k|k-1}}\frac{r_{k|k-1}^{\left(i\right)}\sum_{j=1}^{n_{k-1}^{i}}w_{k|k-1}^{i,m,j}\frac{\sum_{j=1}^{n_{k-1}^{i}}w_{k|k-1}^{i,j}\delta_{l^{i,j}}(c^{m})}{\sum_{j=1}^{n_{k-1}^{i}}w_{k|k-1}^{i,j}}p_{D,k}g_{k}^{k}(x_{k})g_{k}^{c}(x_{k},c^{m})\delta_{x_{k|k-1}^{i,m,j}}(x_{k})}{1-r_{k|k-1}^{\left(i\right)}}}{\sum_{i=1}^{M_{k|k-1}}\frac{r_{k|k-1}^{\left(i\right)}\frac{\sum_{j=1}^{n_{k-1}^{i}}w_{k|k-1}^{i,j}\delta_{l^{i,j}}(c^{m})}{\sum_{j=1}^{n_{k-1}^{i}}w_{k|k-1}^{i,j}}\sum_{j=1}^{n_{k-1}^{i}}w_{k|k-1}^{i,m,j}p_{D,k}g_{k}(x_{k|k-1}^{i,m,j})g_{k}(x_{k|k-1}^{i,m,j})}{1-r_{k|k-1}^{\left(i\right)}}}\\ & =\frac{\sum_{i=1}^{M_{k|k-1}}\frac{\sum_{j=1}^{n_{k-1}^{i}}r_{k|k-1}^{\left(i\right)}w_{k|k-1}^{i,j}\delta_{l^{i,j}}(c^{m})p_{D,k}g_{k}^{k}(x_{k})g_{k}^{c}(x_{k},c^{m})\delta_{x_{k|k-1}^{i,m,j}}(x_{k})}{1-r_{k|k-1}^{\left(i\right)}}}{\sum_{i=1}^{M_{k|k-1}}\frac{\sum_{j=1}^{n_{k-1}^{i}}r_{k|k-1}^{\left(i\right)}w_{k|k-1}^{i,j}\delta_{l^{i,j}}(c^{m})p_{D,k}g_{k}(x_{k|k-1}^{i,m,j})g_{k}(x_{k|k-1}^{i,m,j})}{1-r_{k|k-1}^{\left(i\right)}}} \end{array}$$

(A14) $$\begin{array}{ll} p_{U,k}^{\left(i\right)}(c^{m}|Z^{k}) & =\frac{\sum_{i=1}^{M_{k|k-1}}\frac{r_{k|k-1}^{\left(i\right)}p_{k|k-1}^{\left(i\right)}(c^{m}|Z^{k-1})\langle p_{k|k-1}^{\left(i\right)}(x_{k}|c^{m},Z^{k-1}),p_{D,k}g_{k}^{k}(x_{k})g_{k}^{c}(x_{k},c^{m})\rangle }{1-r_{k|k-1}^{\left(i\right)}}}{\sum_{i=1}^{M_{k|k-1}}\frac{r_{k|k-1}^{\left(i\right)}}{1-r_{k|k-1}^{\left(i\right)}}\sum_{m=1}^{n_{c}}p_{k|k-1}^{\left(i\right)}(c^{m}|Z^{k-1})\langle p_{k|k-1}^{\left(i\right)}(x_{k}|c^{m},Z^{k-1}),p_{D,k}g_{k}^{k}(x_{k})g_{k}^{c}(x_{k},c^{m})\rangle }\\ & =\frac{\sum_{i=1}^{M_{k|k-1}}\frac{r_{k|k-1}^{\left(i\right)}\frac{\sum_{j=1}^{n_{k-1}^{i}}w_{k|k-1}^{i,j}\delta_{l^{i,j}}(c^{m})}{\sum_{j=1}^{n_{k-1}^{i}}w_{k|k-1}^{i,j}}\langle \sum_{j=1}^{n_{k-1}^{i}}w_{k|k-1}^{i,m,j}\delta_{x_{k|k-1}^{i,m,j}}(x_{k}),p_{D,k}g_{k}^{k}(x_{k})g_{k}^{c}(x_{k},c^{m})\rangle }{1-r_{k|k-1}^{\left(i\right)}}}{\sum_{i=1}^{M_{k|k-1}}\frac{r_{k|k-1}^{\left(i\right)}}{1-r_{k|k-1}^{\left(i\right)}}\sum_{m=1}^{n_{c}}\frac{\sum_{j=1}^{n_{k-1}^{i}}w_{k|k-1}^{i,j}\delta_{l^{i,j}}(c^{m})}{\sum_{j=1}^{n_{k-1}^{i}}w_{k|k-1}^{i,j}}\langle \sum_{j=1}^{n_{k-1}^{i}}w_{k|k-1}^{i,m,j}\delta_{x_{k|k-1}^{i,m,j}}(x_{k}),p_{D,k}g_{k}^{k}(x_{k})g_{k}^{c}(x_{k},c^{m})\rangle }\\ & =\frac{\sum_{i=1}^{M_{k|k-1}}\frac{r_{k|k-1}^{\left(i\right)}\sum_{j=1}^{n_{k-1}^{i}}w_{k|k-1}^{i,j}\delta_{l^{i,j}}(c^{m})p_{D,k}g_{k}^{k}(x_{k|k-1}^{i,m,j})g_{k}^{c}(x_{k|k-1}^{i,m,j})}{1-r_{k|k-1}^{\left(i\right)}}}{\sum_{i=1}^{M_{k|k-1}}\frac{r_{k|k-1}^{\left(i\right)}}{1-r_{k|k-1}^{\left(i\right)}}\sum_{m=1}^{n_{c}}\sum_{j=1}^{n_{k-1}^{i}}w_{k|k-1}^{i,j}\delta_{c^{i,j}}(m)p_{D,k}g_{k}^{k}(x_{k|k-1}^{i,m,j})g_{k}^{c}(x_{k|k-1}^{i,m,j})}\\ & =\frac{\sum_{i=1}^{M_{k|k-1}}\frac{r_{k|k-1}^{\left(i\right)}\sum_{j=1}^{n_{k-1}^{i}}w_{k|k-1}^{i,j}\delta_{l^{i,j}}(c^{m})p_{D,k}g_{k}^{k}(x_{k|k-1}^{i,m,j})g_{k}^{c}(x_{k|k-1}^{i,m,j})}{1-r_{k|k-1}^{\left(i\right)}}}{\sum_{i=1}^{M_{k|k-1}}\frac{r_{k|k-1}^{\left(i\right)}}{1-r_{k|k-1}^{\left(i\right)}}\sum_{j=1}^{n_{k-1}^{i}}w_{k|k-1}^{i,j}p_{D,k}g_{k}^{k}(x_{k|k-1}^{i,j})g_{k}^{c}(x_{k|k-1}^{i,j})} \end{array}$$
